# Multi-locus genome-wide association study for phosphorus use efficiency in a tropical maize germplasm

**DOI:** 10.3389/fpls.2024.1366173

**Published:** 2024-08-23

**Authors:** Douglas Mariani Zeffa, Luiz Perini Júnior, Rafael de Assis, Jéssica Delfini, Antoni Wallace Marcos, Alessandra Koltun, Viviane Yumi Baba, Leonel Vinícius Constantino, Renan Santos Uhdre, Alison Fernando Nogueira, Vania Moda-Cirino, Carlos Alberto Scapim, Leandro Simões Azeredo Gonçalves

**Affiliations:** ^1^ Departamento de Agronomia, Universidade Estadual de Maringá, Maringá, Paraná, Brazil; ^2^ Departamento de Agronomia, Universidade Estadual de Londrina, Londrina, Paraná, Brazil; ^3^ Departamento de Biologia, Universidade Estadual de Londrina, Londrina, Paraná, Brazil; ^4^ Área de Melhoramento Genético e Propagação Vegetal, Instituto de Desenvolvimento Rural do Paraná, Londrina, Paraná, Brazil

**Keywords:** *Zea mays* L., GWAS, PUE, root architecture system, associative mapping

## Abstract

Phosphorus (P) is an essential macronutrient for maize (*Zea mays* L.) growth and development. Therefore, generating cultivars with upgraded P use efficiency (PUE) represents one of the main strategies to reduce the global agriculture dependence on phosphate fertilizers. In this work, genome-wide association studies (GWAS) were performed to detect quantitative trait nucleotide (QTN) and potential PUE-related candidate genes and associated traits in greenhouse and field trials under contrasting P conditions. The PUE and other agronomy traits of 132 maize inbred lines were assessed in low and normal P supply through the greenhouse and field experiments and Multi-locus GWAS was used to map the associated QTNs. Wide genetic variability was observed among the maize inbred lines under low and normal P supply. In addition, we confirm the complex and quantitative nature of PUE. A total of 306 QTNs were associated with the 24 traits evaluated using different multi-locus GWAS methods. A total of 186 potential candidate genes were identified, mainly involved with transcription regulator, transporter, and transference activity. Further studies are still needed to elucidate the functions and relevance of these genes regarding PUE. Nevertheless, pyramiding the favorable alleles pinpointed in the present study can be considered an efficient strategy for molecular improvement to increase maize PUE.

## Introduction

The significant increase in maize yield is mainly attributed to genetic improvement, crop management, and fertilizer use (Byerlee, 2020). On the other hand, the dependence of modern agriculture on mineral fertilizers is alarming. The excessive and indiscriminate use of fertilizers has caused severe environmental problems such as water eutrophication, soil acidification, and air pollution (Seleiman et al., 2021). More than 20 million tons of phosphate fertilizers are applied in agriculture annually, and the global demand will be ~25 million tons by 2050 (Bindraban et al., 2020). Moreover, phosphate rocks are a finite resource, and their global reserves could be depleted in the next 300–400 years (Glaser and Lehr, 2019).

In Brazil, agriculture is extremely dependent on imported phosphate fertilizers, and their use has increased over the years (Withers et al., 2018). Most Brazilian soils are high-phosphorus (P) fixing soils, intensely weathered and rich in (hydro)oxides of iron and aluminum (Rodrigues et al., 2021). Thus, large amounts of phosphate fertilizers are needed to overcome the rapid immobilization of inorganic P (Pi) in these regions. In addition, the expansion of Brazilian agricultural lands by the conversion of degraded pastures or native savannas (Cerrado region) will require considerable amounts of phosphate fertilizers (Withers et al., 2018). In this context, developing cultivars with greater P use efficiency (PUE) represents one of the main strategies to reduce Brazilian agriculture's dependence on phosphate fertilizers and reduce the demand for P input (Pavinato et al., 2020) as well as one step further into sustainability.

Plants have developed several adaptive mechanisms to enhance Pi availability in the soil, along with better uptake, translocation, and use under limiting conditions of this nutrient (Wang et al., 2019a, 2021). For instance, root exudation of phosphatases and organic acids is key in improving soil Pi availability (Wang and Lambers, 2020). Plants can also modify root system architecture to increase Pi uptake under P-limiting conditions (Iqbal et al., 2020). Moreover, high-affinity Pi transporters are abundantly produced under P-deficient conditions to raise Pi root uptake and redistribution within the plant (Wang et al., 2021). Additionally, several alternative metabolic pathways and lipid membrane remodeling play a leading role in redistributing Pi from senescent to developing tissues to allow maximum biomass production (Dissanayaka et al., 2021).

Among the molecular improvement methods, genome-wide association studies (GWAS) have been widely adopted to identify quantitative trait nucleotides (QTN) in various crops worldwide (Li et al., 2019a; Cortes et al., 2021). Because PUE is a highly complex trait, strongly influenced by the environment and controlled by several genes with minor effects (Parentoni et al., 2010; Mendes et al., 2014; Meirelles et al., 2016; Bernardino et al., 2019), the use of multi-locus GWAS methods is better suited to dissect the genetic architecture of this trait in plants (Zhang et al., 2019). Multi-locus GWAS methods rely on a random-SNP-effect model where no multiple correction test is required. There are generally two steps in these models. Initially, a reduced number of molecular markers is selected using different algorithms. These markers are then used in multi-locus models to distinguish true signals (Zhang et al., 2019). Recently, several multi-locus GWAS methods have been developed and have shown increased detection power and accuracy to estimate QTN effects when compared to single-locus GWAS methods (Zhang et al., 2020).

Many quantitative trait loci (QTL) have already been identified in maize for low P tolerance, mainly related to root architecture system and biomass accumulation during early development stages (Zhang et al., 2014; Sahito et al., 2020). On the other hand, few studies were carried out in the field. Among them, studies that quantified PUE and its components are even scarcer, focusing more on grain yield components (Xu et al., 2018) and on root system architecture (Gu et al., 2016; Wang et al., 2019). In this sense, the objectives of the present study were: i) to estimate the genetic variability in tropical inbred maize lines for PUE-related traits under contrasting P conditions, ii) verify possible correlations between the traits evaluated in the greenhouse and field, iii) detect genomic regions associated with PUE-associated traits, and iv) identify potential candidate genes to improve maize PUE.

## Materials and methods

### Genetic material

A total of 132 tropical maize inbred lines were evaluated, composed of 77 field corn and 55 popcorn lines from the Maize Breeding Program of the State University of Maringá (UEM), Maringá, Brazil. These inbred lines represent part of the genetic variability present in the main hybrids and varieties of field corn and popcorn cultivated in Brazil. Seed samples were obtained from the UEM Germplasm Bank and later multiplied in the 2017-2018 summer season to standardize seed germination. Information on the origin of the maize lines is presented in **Supplementary Table 1**.

### Greenhouse trials

Inbred lines were evaluated under controlled conditions in a greenhouse at the Agronomy Department of the State University of Londrina (UEL) in Londrina, Paraná, Brazil (23°17'34”S, 51°10'24”W, and 550 m altitude) in September 2019. Two independent hydroponic systems were developed for low (2.5 μM) and normal P (250 μM) conditions using Magnavaca nutrient solution (Magnavaca et al., 1987). Each hydroponic system consisted of eight 28-L polyethylene boxes (58 × 40 × 16.5 cm). The hydroponic boxes were interconnected and connected to a 300-L reservoir, that is, the same nutrient solution ran throughout the entire system. The pH of the nutrient solution was maintained at 5.8 ± 0.2 and permanently aerated by a pressurizing water pump (Model Pl400P 40 MCA, Lorenzetti Ltda, Campinas, Brazil).

Seeds were disinfested by immersion in 95% ethanol solution (v/v) for 30 s followed by immersion in 5% H_2_O_2_ solution (v/v) for 10 min and six washes with sterile deionized water. The seeds were then placed on filter paper (Germtest^TM^, 24 × 33 × 0.02 cm) (Cienlab Ltda., Campinas, Brazil), moistened with distilled water, and placed in a growth chamber at a temperature of 27 ± 2°C and 70% relative humidity. Seven days after sowing (at development stage V1), uniform seedlings were selected and transplanted to hydroponic systems after removing the endosperm to eliminate seed reserves. The experiment was carried out using a completely randomized design with three repetitions. After 12 days of growth in nutrient solution (stage V2), the inbred lines root system was digitized in a 600 dpi image using an Epson L3110 scanner (Seiko Epson Corp., California, USA), and the root architecture traits were assessed using the GiaRoots software (Galkovskyi et al., 2012).

### Field trials

Inbred lines were evaluated at the School Farm of UEL (23°17'34”S, 51°10'24”W, and 550 m altitude) during the 2018–2019 summer and 2019 fall/winter seasons. This region has a humid subtropical climate and soil classified as Dystroferric Red Latosol. Two distinct areas were selected to conduct the experiments under low and normal P conditions, carried out in the same areas during the two seasons. P (Mehlich 1) contents in the areas ranged from 4.87–5.11 mg dm^3^ (low P) to 17.32–19.32 mg dm^3^ (normal P). The soil physicochemical analyses and other traits of the experimental areas are presented in **Supplementary Table 2**.

The experiment was set in a randomized complete block design with three repetitions. Plots consisted of a 4 m long line, 0.45 m spacing between lines, and 0.20 m between plants. Two levels of phosphate fertilization were used in each environment, totaling four independent experiments. Before planting, normal P environments were fertilized with 120 kg P_2_O_5_ ha^–1^, 40 kg K_2_O ha^–1^, and 40 kg N ha^–1^, while low P environments only received 40 kg K_2_O ha^–1^ and 40 kg N ha^–1^. The topdressing nitrogen (N) fertilization was carried out using 180 kg N ha^–1^ applied at stage V6, while the other cultural practices were performed according to crop demand. At physiological maturation (stage R6), three uniform and representative plants from each plot were collected for phenotypic evaluations.

### Phenotyping under greenhouse and field conditions

The detailed description of the 24 traits evaluated in the greenhouse and field experiments is presented in **Table 1**. To determine the P content in plant tissues, samples were oven-dried at 60°C for 72 h and milled in Willey-type knife mill MA340 (Piracicaba, São Paulo, Brazil). Then 0.1 g aliquots were digested in nitroperchloric solution (HNO_3_:HClO_4_) according to Malavolta et al. (1989). P content was determined by the molybdenum blue spectrophotometric method (Pradhan and Pokhrel, 2013), reading the samples in an Agilent 8453 spectrophotometer (Agilent Technologies, California, USA) at 660 nm.

**Table 1:** Description of the 24 traits evaluated in 132 tropical corn lines under greenhouse and field conditions.

Traits	Unity	Description
Greenhouse evaluations		
Shoot biomass (SB_g)	mg	Shoot biomass of samples oven-dried at 60°C for 72 h
Root biomass (RB)	mg	Root biomass of samples oven-dried at 60°C for 72 h
Total dry biomass (TB)	mg	Sum of dry shoot and root biomass
Root superficial area (RSA)	cm^2^	Root superficial area measured by the GiaRoots software (Galkovskyi et al., 2012)
Root volume (RV)	cm^3^	Root volume measured by the GiaRoots software (Galkovskyi et al., 2012)
Root average diameter (RD)	mm	Root average diameter measured by GiaRoots software (Galkovskyi et al., 2012)
Number of total roots (NR)	number	Root total number measured by the GiaRoots software (Galkovskyi et al., 2012)
Root length (RL)	cm	Root total length by the GiaRoots software (Galkovskyi et al., 2012)
Phosphorus content in the shoot (PS_g)	mg g^–1^	Shoot phosphorus content
Phosphorus uptake efficiency (PUpE_g)	mg mg^–1^	Ratio between plant total P total and P available for the plant (Moll et al., 1982)
Phosphorus utilization efficiency (PUtE_g)	mg mg^–1^	Ratio between shoot dry biomass and plant total P (Moll et al., 1982)
Phosphorus use efficiency (PUE_g)	mg mg^–1^	Ratio between shoot dry biomass and P available for the plant (Moll et al., 1982)
Field evaluations		
Plant height	cm	Measured from the soil until the flag leaf insertion
Ear height (EH)	cm	Measured from the soil until the main ear insertion
Ear length (EL)	cm	Average ear length measured with a digital caliper
Ear diameter (ED)	mm	Average ear diameter measured with a digital caliper
Shoot biomass (SB_f)	g	Shoot biomass of samples oven-dried at 60°C for 72 h
Harvest index (HI)	%	Ratio between grain yield and shoot dry biomass
Phosphorus harvest index (PHI)	%	Ratio between grain phosphorus content and shoot phosphorus content
Phosphorus content in the grain (PG)	g kg^–1^	Grain phosphorus content
Phosphorus content in the shoot (PS_f)	g kg^–1^	Shoot phosphorus content
Phosphorus uptake efficiency (PUpE_f)	g g^–1^	Ratio between plant total P and P available for the plant (Moll et al., 1982)
Phosphorus utilization efficiency (PUtE_f)	g g^–1^	Ratio between grain yield and plant total P (Moll et al., 1982)
Phosphorus use efficiency (PUE_f)	g g^–1^	Ratio between grain yield and P available for the plant (Moll et al., 1982)

### Deviance analysis and genetic parameters

Data were analyzed using the best linear unbiased predictor (BLUP) and restricted maximum likelihood (REML) methods by the software Selegen–REML/BLUP (Resende, 2016) and R version 3.6.0 (https://www.r-project.org) via the 'lme4' package. The deviance analyses (ANADEV) of the traits obtained from the greenhouse and field trials were carried out using the following mathematical models, respectively:


$$y_{im}=µ+G_{i}+P_{m}+GP_{im}{+}_{\epsilon im}$$

Where *µ* is the overall mean, *G*_i_ is the random effect of the i-th genotype, *P*_m_ is the fixed effect of the m-th level of P, *GP*_im_ is the random effect of the genotype × P level interaction, and *ϵ*_im_ ~ N(0, σ²) is the random effect of the error associated with each experimental unit.


$$y_{ijkm}=µ+G_{i}+B_{j/k/m}+S_{k}+P_{m}+GS_{ik}+GP_{im}+SP_{km}+GSP_{ikm}{+}_{\epsilon ijkm}$$

Where µ is the overall mean, *G*_i_ is the random effect of the i-th genotype, *B*_j/k/m_ is the random effect of the j-th block within the k-th season and within the m-th P level, *S*_k_ is the fixed effect of the k-th season, *P*_m_ is the fixed effect of the m-th P level, *GS*_ik_ is the random effect of the genotype × season interaction, *GP*_im_ is the random effect of the genotype × P level interaction, *SP*_km_ is the fixed effect of the yield × P level interaction, *GSP*_ikm_ is the random effect of the genotype × yield × P level interaction, and *ϵ*_ijkn_ ~ N(0, σ²) is the random effect of the error associated with each experimental unit.

The significance of the ANADEV random effects was verified by the likelihood ratio test (Resende, 2016). The heritability in the broad sense (h^2^) of the traits evaluated in the greenhouse and field were estimated, respectively, using the following formulas: $h^{2}=\frac{{\hat{\text{σ}}}_{\text{g}}^{2}}{{\hat{\text{σ}}}_{\text{g}}^{2}\;+{\;\hat{\text{σ}}}_{\text{e}}^{2}}$ and $h^{2}=\frac{{\hat{\text{σ}}}_{\text{g}}^{2}}{{\hat{\text{σ}}}_{\text{g}}^{2}\;+\;\frac{{\hat{\text{σ}}}_{\text{gs}}^{2}}{s}\;+\;\frac{{\hat{\text{σ}}}_{\text{e}}^{2}}{rs}}$ , where ${\hat{\text{σ}}}_{\text{g}}^{2}$ is the genotypic variance, ${\hat{\text{σ}}}_{\text{gs}}^{2}$ is the variance of the genotype × season interaction, ${\hat{\text{σ}}}_{\text{e}}^{2}$ is the residual variance, *r* is the number of repetitions in each season, and *s* is the number of seasons. The selective accuracy (Ac) was obtained as follows: $Ac=\sqrt{\frac{1-\text{PEV}}{{\hat{\text{σ}}}_{\text{g}}^{2}}}$ , where *PEV* is the variance of the prediction error of the genotypic values, and ${\hat{\text{σ}}}_{\text{g}}^{2}$ is the genotypic variance.

### Correlations and principal component analysis

Correlations between traits and principal component analyses (PCA) were performed using BLUP means. The significance of the estimates was verified using the t-test at a 5% probability level (α = 0.05). The correlation estimates were visualized using the correlation network approach. These analyses were performed by the 'qgraph' and 'ggbiplot' packages of the R software version 3.6.0.

### Genotyping-by-sequencing

Genomic DNA was initially isolated from leaf tissues of inbred lines, as established by Coan et al. (2018). DNA samples were then sent to the Genomic Diversity Institute at Cornell University for genotyping-by-sequencing (GBS) according to the protocol described by Elshire et al. (2011). Sequencing was performed in the Illumina HiSeq 2000 sequencer (Illumina Inc., San Diego, USA). The sequences were deposited in the European Variation Archive (EVA) (GCF_902167145.1).

Sequencing data were analyzed using the Tassel 5.0 GBS v2 software (Glaubitz et al., 2014). The sequences obtained were aligned to the reference genome of *Zea mays* L. version AGPV3 (B73 RefGen v3) obtained from the MaizeGDB database (https://www.maizegdb.org). SNP markers quality control was performed using the VCFtools software version 0.1.15 (Danecek et al., 2011), removing the SNPs through the following criteria: i) non-biallelic, ii) minor allele frequency (MAF) less than 5%, iii) inbreeding coefficient less than 90%, and iv) missing data greater than 90%. Heterozygous SNPs were treated as missing data since the evaluated lines are highly inbred and, thus, these SNPs may come from sequencing errors. Missing data were imputed with the hidden Markov model (HMM) using the Beagle software version 5.0 (Browning et al., 2018). After filtering, a total of 273,775 high-quality SNPs were retained for subsequent analyses (**Supplementary Figure 1**).

### Population structure and kinship matrix

The population genetic structure was inferred using the Bayesian clustering model through the software Structure version 2.3.4 (Pritchard et al., 2000) based on the method described by Evanno et al. (2005). One hundred thousand MCMC (Monte Carlo Markov chain) iterations, a burn-in period of 10,000 iterations, an admixture model, and correlated allelic frequencies were used. Subgroup values (Δ*K*) between one and ten were tested, with ten independent interactions for each *K* value. The ideal number of *K* was determined using the Structure Harvester software version 0.6.92 (Earl, 2012).

PCA was performed using the Tassel software version 5.2.48 (Bradbury et al., 2007). Additionally, the Euclidean distance between the inbred lines was calculated, and the UPGMA (unweighted pair group method using arithmetic averages) hierarchical grouping was performed. These analyses were performed using the R software version 3.6.0 via the packages 'SNPRelate', 'factoextra', and 'ggplot2'. The kinship matrix was calculated based on the centralized identity-by-state (IBS) method (Endelman and Jannink, 2012) using the Tassel software version 5.2.48 (Bradbury et al., 2007).

### Linkage disequilibrium analysis

Linkage disequilibrium (LD) analysis was performed using the 'LDcorSV' package of the R software version 3.6.0. LD was estimated on all chromosomes simultaneously and individually. In addition to the conventional measure of r^2^, corrected r^2^ was estimated by: i) population structure (*r^2^Q*), ii) kinship matrix (*r^2^*_K_), and iii) population structure plus kinship matrix (*r^2^QK*) (Mangin et al., 2012). The results of the Bayesian clustering and IBS method were used to correct the population structure and kinship matrix, respectively, as presented above. LD was adjusted using the nonlinear regression method proposed by Hill and Weir (1988) using the *nls* function of the R software version 3.6.0. The LD decay was defined by the distance at which half of the maximum LD decayed (LD half decay). This estimate indicates the initial slope of the LD decay, and it was considered the most consistent in the comparative study conducted by Vos et al. (2017).

### Multi-locus GWAS

For multi-locus GWAS analyses, least square means (lsmeans) estimated from the lsmeans function of PROC GLM in SAS software version 9.0 (SAS Institute, Cary, USA) were used. Multi-locus GWAS analyses were performed using eight datasets: i) 2018-2019 summer season at low P (LP_18), ii) 2018-2019 summer season under normal P (NP_18), iii) 2019 fall/winter season in low P (LP_19), iv) 2019 fall/winter season in high P (NP_19), v) combination of 2018–2019 and 2019 seasons in low P (LP_C), vi) combination of 2018–2019 and 2019 seasons under normal P (NP_C), vii) greenhouse at low P (LP_G), and vii) greenhouse in normal P (NP_G).

Five multi-locus GWAS methods implemented in the ‘mrMLM.GUI’ package (Zhang et al., 2020) of the R software version 3.6.0 were used: i) mrMLM (Wang et al., 2016), ii) FASTmrMLM (Tamba and Zhang, 2018), iii) FASTmrEMMA (Wen et al., 2018), iv) ISIS EM-BLASSO (Tamba et al., 2017), and v) pLARmEB (Zhang et al., 2017). The population structure (*Q*) and kinship matrix (*K*) were included in the tested models to minimize the identification of false-positive associations and increase the statistical analysis power. Critical values for significant associations were LOD (logarithm of odds) ≥ 3 (or *P* = 0.0002) for all methods. To obtain more accurate results, only the QTNs detected by at least three different methods were considered truly significant and, later, used in the search for candidate genes.

### Favorable alleles

The QTNs detected had their favorable alleles identified, that is, the favorable alleles that cause positive effects on the traits. Then, a heatmap based on Ward's method and Euclidean distance was performed to group the inbred maize lines into clusters with different amounts of favorable alleles. In addition, box plots were made to verify if the pyramiding of these favorable alleles would result in higher PUE and its components. These analyses were performed using R software version 3.6.0. through the 'pheatmap' and 'ggplot2' packages.

### Candidate genes

Candidate genes were selected based on the reference genome of *Zea mays* L. version AGPV3 (B73 RefGen v3) obtained from the MaizeGDB database (https://www.maizegdb.org). The search radius of candidate genes was established based on the results obtained from the LD half decay. The classical genes or those with known functions in maize were annotated using the MaizeGDB database. Additionally, the molecular functions of all candidate genes were annotated according to the Gene Ontology (GO) database (http://www.geneontology.org/).

## Results

### Deviance analysis and genetic parameters

The deviance analyses showed a significant effect (*P* ≤ 0.01) of genotype and genotype–P level interaction for all traits evaluated in the greenhouse trials (**Table 2**). Low P reduced all characteristics, except for PUtE_g, PUpE_g, and PUE_g. The reductions in these traits ranged from –4.8 (RD) to –65.0% (PS_g), while a high increase was observed for PUtE_g (161.9%), PUpE_g (169.6%), and PUE_g (678.9%) (**Supplementary Figure 2**). The estimates of h^2^ ranged from 0.55 (RV) to 0.74 (RD and RSA) at low P, while under normal P conditions, the estimates varied from 0.63 (RV) to 0.86 (RD). Ac values ranged from 0.74 (RV) to 0.86 (RD and RSA) and 0.86 (RV and SB_g) to 0.92 (RD and RSA) under low and normal P conditions, respectively.

**Table 2:** Mean, standard deviation (SD), broad-sense heritability (h^2^), selective accuracy (Ac), and likelihood ratio test (LRT) for 24 traits evaluated under normal (NP) and low phosphorus (LP) conditions obtained in the greenhouse and field.

Traits^1/^	Means ± SD		ΔP%	h^2^		Ac		LRT^1^			
	LP	NP		LP	NP	LP	NP	G	G×P	G×S	G×P×S
Greenhouse											
RD (mm)	0.38 ± 0.05	0.40 ± 0.03	–4.8	0.74	0.86	0.86	0.92	**	**		
NR (n°)	10.0 ± 2.8	11.6 ± 3.4	–13.8	0.69	0.77	0.83	0.91	**	**		
RSA (cm^2^)	27.5 ± 11.1	37.2 ± 8.3	–26.0	0.74	0.75	0.86	0.92	**	**		
RL (cm)	215.1 ± 11.2	311.2 ± 87.3	–30.8	0.67	0.69	0.81	0.90	**	**		
RV (cm^3^)	0.35 ± 0.14	0.46 ± 0.12	–23.8	0.55	0.72	0.74	0.86	**	**		
SB_g (g)	71.3 ± 22.4	88.1 ± 11.3	–19.0	0.56	0.64	0.75	0.86	**	**		
RB (g)	30.9 ± 8.6	41.7 ± 9.2	–25.9	0.58	0.66	0.76	0.87	**	**		
TB (g)	102.3 ± 30.7	129.8 ± 33.9	–21.1	0.61	0.63	0.78	0.88	**	**		
PS_g_ (mg g^–1^)	0.74 ± 0.12	2.1 ± 0.4	–65.0	0.71	0.79	0.84	0.91	**	**		
PUtE_g (mg mg^–1^)	0.49 ± 0.15	0.18 ± 0.04	161.9	0.64	0.81	0.80	0.89	**	**		
PUpE_g (mg mg^–1^)	1493 ± 268	554 ± 112	169.6	0.63	0.79	0.79	0.88	**	**		
PUE_g (mg mg^–1^)	711.4 ± 220.9	91.3 ± 23.2	678.9	0.67	0.84	0.81	0.91	**	**		
Field											
PH (cm)	129.3 ± 12.9	136.6 ± 14.9	–5.2	0.72	0.81	0.84	0.90	**	*ns*	**	*ns*
EH (cm)	64.1 ± 9.5	69.1 ± 11.2	–7.2	0.68	0.82	0.82	0.91	**	*ns*	**	*
SB_f (g)	163.7 ± 46.4	186.4 ± 37.9	–12.2	0.61	0.74	0.78	0.86	**	*	**	*ns*
ED (mm)	30.7 ± 4.5	32.4 ± 5.0	–5.2	0.61	0.68	0.78	0.82	**	*ns*	**	**
EL (cm)	12.2 ± 1.6	12.8 ± 1.8	–4.8	0.66	0.73	0.81	0.85	**	**	**	*
PS_f (g kg^–1^)	1.3 ± 0.2	1.3 ± 0.3	–5.2	0.51	0.58	0.71	0.76	**	**	**	**
PG (g kg^–1^)	1.9 ± 0.2	3.1 ± 0.3	–37.4	0.48	0.63	0.69	0.79	**	*ns*	**	*ns*
PHI (%)	0.19 ± 0.03	0.31 ± 0.04	–37.5	0.45	0.61	0.67	0.82	**	*ns*	**	*ns*
HI (%)	36.5 ± 16.1	40.7 ± 17.6	–10.3	0.44	0.59	0.66	0.77	**	**	**	**
PUtE_f (g g^–1^)	210.9 ± 35.6	176.5 ± 31.1	19.4	0.46	0.67	0.67	0.81	**	**	**	**
PUpE_f (g g^–1^)	0.102 ± 0.044	0.031 ± 0.013	221.9	0.48	0.65	0.69	0.80	**	**	*	*
PUE_f (g g^–1^)	19.3 ± 3.2	5.4 ± 1.8	256.7	0.49	0.62	0.70	0.78	**	**	**	**

ns, **, and * indicate non-significance, and significance at 1 and 5% probability by the chi-square test, respectively, for the random effects of genotypes (G) and their interactions with phosphorus (P) levels and season (S). Traits are described in detail in **Table 1**.

In the field trials, the deviance analyses presented significant triple interaction (*P* ≤ 0.05) between genotype–season–P level for EH, ED, EL, PS_f, HI, PUtE_f, and PUE_f. For genotype–P level interaction, significant effects (*P* ≤ 0.05) were observed for SB_f, EL, PS_f, HI, PUtE_f, PUpE_f, and PUE_f. All traits assessed showed significant effects of genotype (*P* ≤ 0.01) and genotype-season interaction (*P* ≤ 0.05). Low P led to reduced values for all evaluated traits, except PUtE_f, PUpE_f, and PUE_f. The reductions in trait values ranged from –4.8 (EL) to –37.5% (PHI), while PUtE_f (19.4%), PUpE_f (221.9%), and PUE_f (256.7%) increased greatly. The estimates of h^2^ ranged from 0.44 (HI) to 0.72 (PH) under low P and from 0.52 (PS_f) to 0.82 (EH) under normal P conditions. Ac values ​​ranged from 0.66 (HI) to 0.84 (PH) and 0.76 (PS_f) to 0.91 (EH) under low and normal P conditions, respectively.

### Correlation and principal component analysis

**Figure 1:**
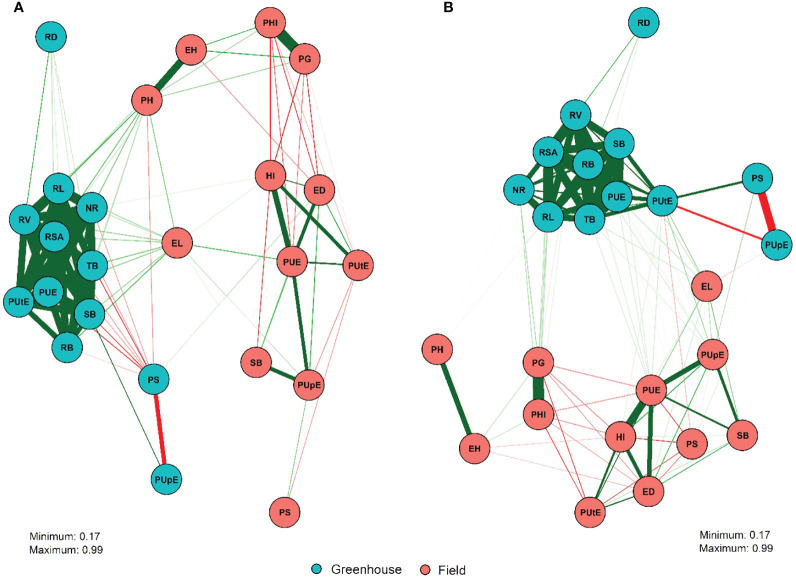
Correlation network among 24 traits evaluated in 132 inbred tropical maize lines under low **(A)** and normal P **(B)** conditions obtained in field and greenhouse trials. A detailed description of the characteristics is presented in **Table 1**. Correlation network between 24 traits evaluated in 132 inbred lines of tropical maize under low (A) and normal P (B) conditions obtained in field and greenhouse trials. Respectfully, the green and red lines represent Pearson estimates positive and negative linear correlations and the thickness of the lines is proportional to the magnitude of the correlation.

The correlations among traits in the greenhouse and field are shown in **Figure 1**. In general, there was a greater correlation between the traits within than between the field and greenhouse trials. However, few differences were observed between low and normal P conditions, mainly due to the magnitudes of the correlation estimates rather than their direction. Strong positive correlations were observed between greenhouse traits under both P conditions, primarily root system-related traits, PUtE_g, and PUE_g. Under field conditions, high and positive correlation estimates were found for PH × EH, PUE_f × HI, PUE_f × PUpE_f, PUE_f × SB_f, PG × PHI, and PUpE_f × SB_f. Conversely, negative correlations were reported for PS_g × PUpE_g in the greenhouse under low and normal P conditions.

**Figure 2:**
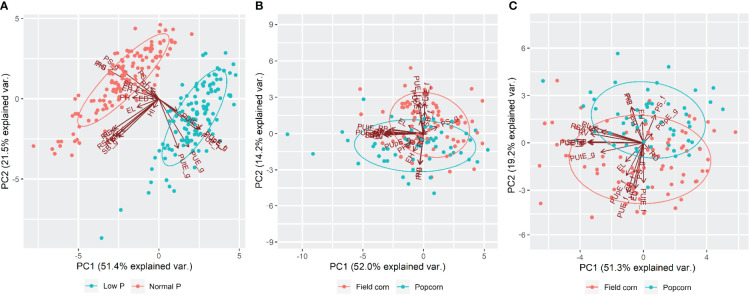
Principal component analysis (PCA) of 132 inbred tropical maize lines evaluated for 24 traits in the greenhouse and field under low versus normal P **(A)**, low P **(B)**, and normal P **(C)** conditions. A detailed description of the characteristics can be found in **Table 1**.

PCA of low versus normal P, low P, and normal P conditions are shown in **Figure 2**. Together, the first two principal components (PC1 and PC2) explained 65.2% (low P), 70.5% (normal P), and 72.9% (low versus normal P) of the total existing variation. There was a clear distinction between low versus normal P, with PUE_g, PUE_f, PUtE_g, PUtE_f, PUpE_g, and PUpE_f vectors associated with low P, while the others were associated with normal P (**Figure 2A**). The field corn and popcorn lines showed distinct collective behavior under both P conditions. At low P, the field corn lines were associated with ED, SB_f, PUtE_f, PUpE_f, and PUE_f vectors, whereas the popcorn lines were mainly linked to the PH, EH, PG, and PHI vectors (**Figure 2B**). Under normal P, the field corn lines were associated with the ED, HI, SB_f, PUtE_f, PUpE_f, and PUE_f vectors, while the popcorn lines were primarily linked with the PH, EH, PG, PHI, and PS_f vectors (**Figure 2C**).

### Population structure

**Figure 3:**
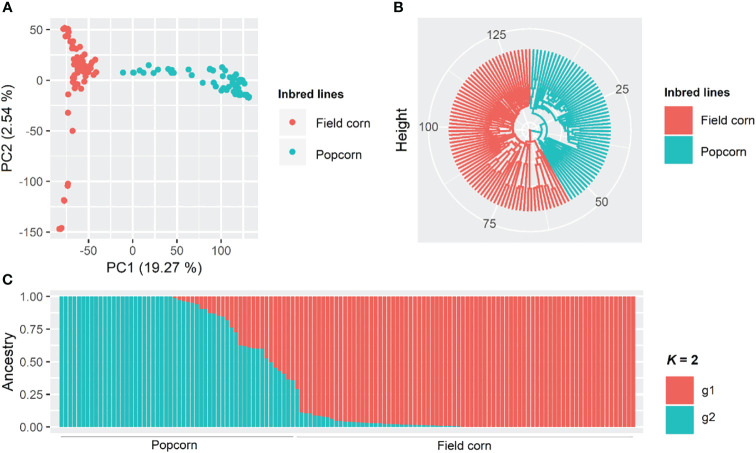
Principal component analysis **(A)**, dendrogram obtained by the UPGMA method (unweighted pair group method using arithmetic averages) through the Euclidean distance **(B)**, and Bayesian clustering considering *K* = 2 **(C)** among 132 tropical maize lines using 273,775 SNP (single nucleotide polymorphism) markers.

Based on the PCA, the first two components explained 19.27 and 2.54% of the total existing variation, respectively, and it is possible to observe a distinction between field corn and popcorn lines (**Figure 3A**). Similar results were found by the UPGMA and Bayesian groupings (**Figures 3B, C**). Bayesian analysis classified seven popcorn lines as admixture, with an ancestry coefficient lower than 0.6 for each subpopulation.

### Linkage disequilibrium analysis

LD showed rapid decay and exhibited variations within each chromosome (**Supplementary Figure 3**). Considering all chromosomes, the differences between uncorrected *r^2^* (*r^2^* = 0.16; LD half decay = 1.41 kb) and corrected population structure (*r^2^K* = 0.15; LD half decay = 1.27 kb) were small. On the other hand, *r^2^* was affected by the corrections of the kinship matrix (*r^2^Q* = 0.07; LD half decay = 0.89 kb) and kinship matrix plus population structure (*r^2^KQ* = 0.07; LD half decay = 0.89 kb), presenting similar results (**Supplementary Figure 4**). Regarding individual chromosomes, Chr07 and Chr05 showed the fastest and slowest decay, respectively. From the results obtained from the LD half decay, the search distance for candidate genes was defined as ± 1 kb.

### QTNs identified and method performance

The histograms of the lsmeans used to identify the QTNs are shown in **Supplementary Figures 5, 6**. A total of 5838 QTNs was discovered by the five different multi-locus GWAS methods evaluated (**Supplementary Figure 7**). The pLARmEB method (n = 1710) identified the highest number of QTNs, followed by the FASTmrMLM (n = 1604), ISIS EM-BLASSO (n = 1224), mrMLM (n = 756), and FASTmrEMMA (n = 544) methods. Among these QTNs, 810 were co-detected by two methods, 255 by three, 44 by four, and seven by five. On the other hand, 3242 QTNs were found by only one of the evaluated methods. Thus, only 255 QTNs were considered highly reliable, as they were co-detected by at least three methods and, consequently, were maintained in the present study (**Supplementary Table 3**).

The FASTmrMLM method was the most efficient (95.4%), as 292 of the 306 QTNs were detected by this method, followed by pLARmEB (94.1%), ISIS EM-BLASSO (85.0%), FASTmrEMMA (29.1%), and mrMLM (19.9%). The 306 QTNs discovered are distributed in the ten chromosomes (Chr) of maize, ranging from 15 (Chr08) to 60 QTNs (Chr01) (**Supplementary Figure 8A**). A greater number of QTNs was detected in the field trials (n = 227) compared to greenhouse trials (n = 79) (**Supplementary Figure 8B**). In the greenhouse, 35 and 44 QTNs were found under low and normal P conditions, respectively (**Supplementary Figure 8C**), whereas 118 (low P) and 107 (normal P) QTNs were detected in the field trials (**Supplementary Figure 8D**).

The 79 QTNs identified in the greenhouse were associated with RV (n = 3), PUpE (n = 4), PUE (n = 5), SB (n = 5), NR (n = 6), RD (n = 6), RL (n = 6), PS (n = 7), TB (n = 7), RSA (n = 8), PUtE (n = 9), and RB (n = 13) (**Supplementary Table 3**). The phenotypic variation explained (PVE) by these QTNs ranged from 0.01 to 16.31%. In turn, the 227 QTNs identified in the field were associated with PUE (n = 13), EL (n = 14), ED (n = 16), PG (n = 18), PH (n = 18), EH (n = 19), HI (n = 19), SB (n = 19), PUtE (n = 20), PUpE (n = 22), PS (n = 24), and PHI (n = 25) (**Supplementary Table 3**). The PVE by these QTNs varied from 0.01 to 18.62%.

A total of 16 pleiotropic QTNs were detected in the greenhouse (n = 10) and field (n = 6) trials (**Table 3**). In the greenhouse experiment under low P, pleiotropic QTNs were associated with SB–TB (S2_170934541), PUtE–RL (S4_198262446), and RL–RSA (S5_97898187 and S9_11783675). Furthermore, a pleiotropic QTN (S1_46130565) was detected in three characteristics (RSA, NR, and RL). Under normal P conditions, pleiotropic QTNs associated with PUtE–PS (S1_1159069), RB–TB (S1_151079747), RSA–TB (S3_225847941), and RB–RSA (S5_212662145) were revealed. Moreover, a highly pleiotropic QTN (S10_119011145) was found in four traits simultaneously (PUE, RB, SB, and TB). In the field trials under low P, pleiotropic QTNs were associated with PUE–PUpE (S3_190210784) and HI–PUE (S8_148017105). Under normal P conditions, pleiotropic QTNs were identified in PG–PH (S1_259083391), HI–PUE (S5_10594899), and ED–PUE (S7_7624808).

**Table 3:** Pleiotropic QTNs identified in traits evaluated in greenhouse and field trials under low and normal P conditions.

QTN	Trait^1^	Dataset^2^	Chr	Position (bp)	QTN effect	LOD score	PVE (%)	MAF	Allele	Method^3^
Greenhouse										
S1_1159069	PUtE	NP_G	1	1159069	–0.04 ~ –0.01	3.83 ~ 62.32	0.38 ~ 3.48	0.042	A	2, 4, 5
	PS	NP_G			–0.49 ~ –0.47	8.14 ~ 9.72	0.41 ~ 5.41			2, 4, 5
S1_151079747	RB	NP_G	1	151079747	1.87 ~ 10.23	3.12 ~ 4.85	0.60 ~ 5.42	0.215	A	1, 3, 4, 5
	TB	NP_G			12.00 ~ 18.27	6.33 ~ 12.43	3.05 ~ 7.08			2, 4, 5
S1_46130565	RSA	LP_G	1	46130565	3.42 ~ 4.45	8.65 ~ 9.77	1.32 ~ 7.13	0.450	C	1, 2, 4, 5
	NR	LP_G			0.61 ~ 1.11	4.36 ~ 12.61	2.33 ~ 7.52			2, 4, 5
	RL	LP_G			20.63 ~ 34.97	4.51 ~ 10.95	2.54 ~ 4.15			1, 4, 5
S2_170934541	SB	LP_G	2	170934541	–13.24 ~ –10.73	7.15 ~ 9.96	4.17 ~ 6.35	0.122	G	1, 2, 4, 5
	TB	LP_G			–14.81 ~ –13.91	6.27 ~ 7.32	1.88 ~ 4.51			2, 4, 5
S3_225847941	RSA	NP_G	3	225847941	–6.70 ~ –4.63	4.88 ~ 9.39	2.73 ~ 5.73	0.118	G	2, 4, 5
	TB	NP_G			–23.44 ~ –10.87	3.39 ~ 12.04	1.57 ~ 7.30			2, 4, 5
S4_198262446	PUtE	LP_G	4	198262446	–0.10 ~ –0.04	6.86 ~ 28.04	0.93 ~ 5.93	0.070	G	2, 4, 5
	RL	LP_G			–98.96 ~ –48.96	5.20 ~ 16.43	3.79 ~ 15.50			2, 4, 5
S5_212662145	RB	NP_G	5	212662145	–5.37 ~ –3.64	3.50 ~ 11.80	2.22 ~ 4.58	0.163	T	1, 2, 4, 5
	RSA	NP_G			–6.75 ~ –3.90	4.98 ~ 10.09	2.50 ~ 7.51			1, 2, 4, 5
S5_97898187	RL	LP_G	5	97898187	20.35 ~ 21.37	4.19 ~ 4.77	0.77 ~ 2.03	0.264	A	2, 4, 5
	RSA	LP_G			2.51 ~ 3.65	4.84 ~ 7.24	0.84 ~ 3.76			2, 4, 5
S9_11783675	RL	LP_G	9	11783675	–51.19 ~ –38.40	4.31 ~ 6.88	0.79 ~ 4.14	0.068	C	2, 4, 5
	RSA	LP_G			–8.93 ~ –5.92	7.16 ~ 8.92	1.18 ~ 7.39			2, 4, 5
S10_119011145	PUE	NP_G	10	119011145	–12.46 ~ –10.23	3.54 ~ 6.73	3.79 ~ 5.63	0.189	C	1, 2, 5
	RB	NP_G			–6.42 ~ –4.86	4.71 ~ 12.12	4.44 ~ 7.75			2, 4, 5
	SB	NP_G			–18.91 ~ –9.25	4.21 ~ 10.74	2.09 ~ 13.88			1, 2, 4, 5
	TB	NP_G			–17.08 ~ –10.96	4.91 ~ 6.20	2.31 ~ 5.62			1, 2, 5
Field										
S1_259083391	PG	NP_18	1	259083391	–0.19 ~ –0.10	3.20 ~ 7.93	1.24 ~ 4.47	0.055	C	2, 4, 5
	PH	NP_18			3.41 ~ 5.39	3.82 ~ 5.12	1.34 ~ 3.35			2, 4, 5
S3_190210784	PUE	LP_C	3	190210784	0.90 ~ 4.78	3.59 ~ 10.56	0.93 ~ 6.52	0.279	G	2, 3, 4, 5
	PUpE	LP_C			0.00 ~ 0.00	3.13 ~ 61.52	0.56 ~ 0.80			2, 4, 5
S5_10594899	HI	NP_C	5	10594899	–6.50 ~ –6.06	6.38 ~ 9.59	4.88 ~ 5.62	0.077	C	2, 4, 5
	PUE	NP_C			–0.97 ~ –0.70	6.64 ~ 7.00	2.46 ~ 4.81			2, 4, 5
S7_7624808	ED	NP_C	7	7624808	0.88 ~ 1.15	7.19 ~ 8.53	2.50 ~ 4.28	0.157	G	1, 2, 5
	PUE	NP_C			0.50 ~ 0.91	4.44 ~ 10.44	2.29 ~ 7.83			2, 4, 5
S8_148017105	HI	LP_19	8	148017105	3.03 ~ 10.42	3.52 ~ 6.12	0.97 ~ 5.29	0.484	C	2, 3, 5
	PUE	LP_19			1.30 ~ 4.12	4.99 ~ 6.34	0.56 ~ 6.55			2, 3, 5
S10_127834511	HI	NP_C	10	127834511	5.18 ~ 6.38	8.69 ~ 8.95	5.12 ~ 7.77	0.116	C	2, 4, 5
	PUE	NP_18			0.88 ~ 1.03	4.57 ~ 6.44	1.00 ~ 2.52			2, 4, 5

^1^Details about the traits are presented in **Table 1**.

^2^2018 season under low P (LP_18), 2018 season in normal P (NP_18), 2019 season under low P (LP_19), 2019 season in high P (NP_19), the combination of 2018 and 2019 seasons in low P (LP_C), the combination of 2018 and 2019 seasons in normal P (NP_C), greenhouse under low P (LP_G), and greenhouse under normal P (NP_G).

^3^1 – mrMLM (Wang et al., 2016), 2 – FASTmrMLM (Tamba and Zhang, 2018), 3 – FASTmrEMMA (Wen et al., 2018), 4 – ISIS EM-BLASSO (Tamba et al., 2017), and 5 – pLARmEB (Zhang et al., 2017).

### Favorable alleles

The heatmap grouped the inbred maize lines into three distinct groups regarding favorable allele number (**Supplementary Figure 9**). Group 1 was composed of 41 lines, characterized by having the smallest number of favorable alleles in the field experiments, mainly under low P conditions. Groups 2 and 3 were formed by 36 and 55 lines, respectively. In general, the lines in group 2 had the highest numbers of favorable alleles in the greenhouse and field trials in both P conditions. On the other hand, group 3 lines had the lowest numbers of favorable alleles, mainly in the greenhouse experiments. In general, there was no distinction between popcorn and field corn lines concerning the accumulation of favorable alleles.

**Figure 4:**
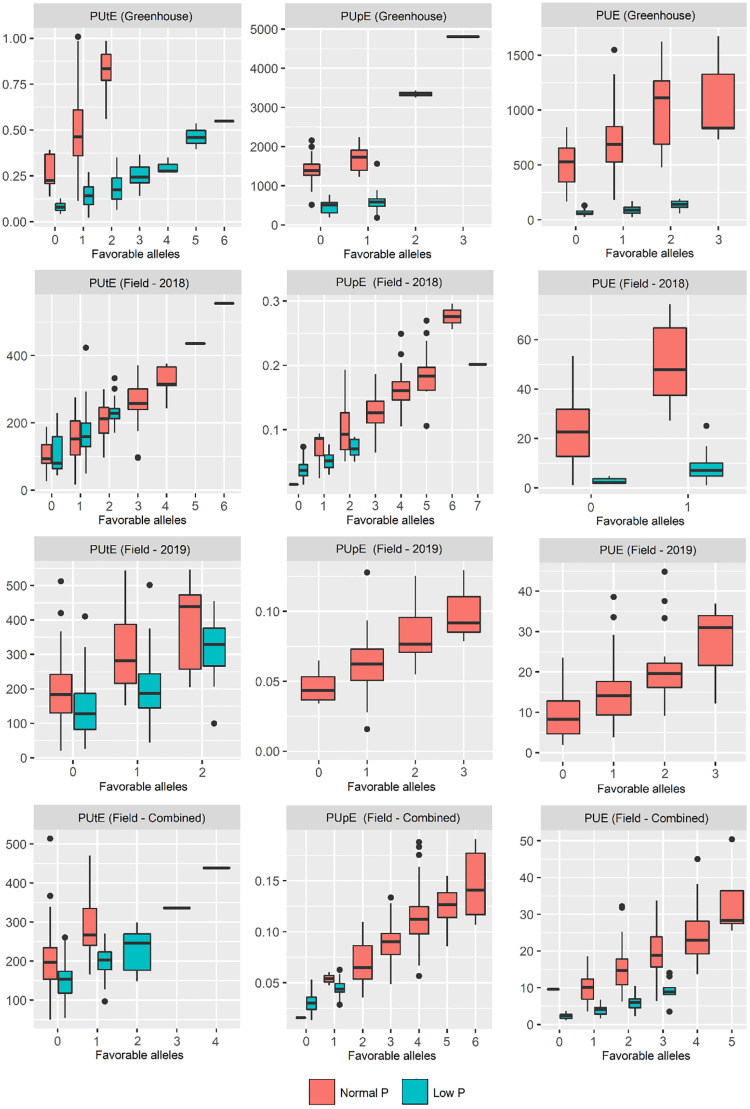
Pyramidization of favorable alleles in inbred maize lines evaluated for traits related to phosphorus utilization efficiency (PUtE), phosphorus uptake efficiency (PUpE), and phosphorus use efficiency (PUE).

**Figure 5:**
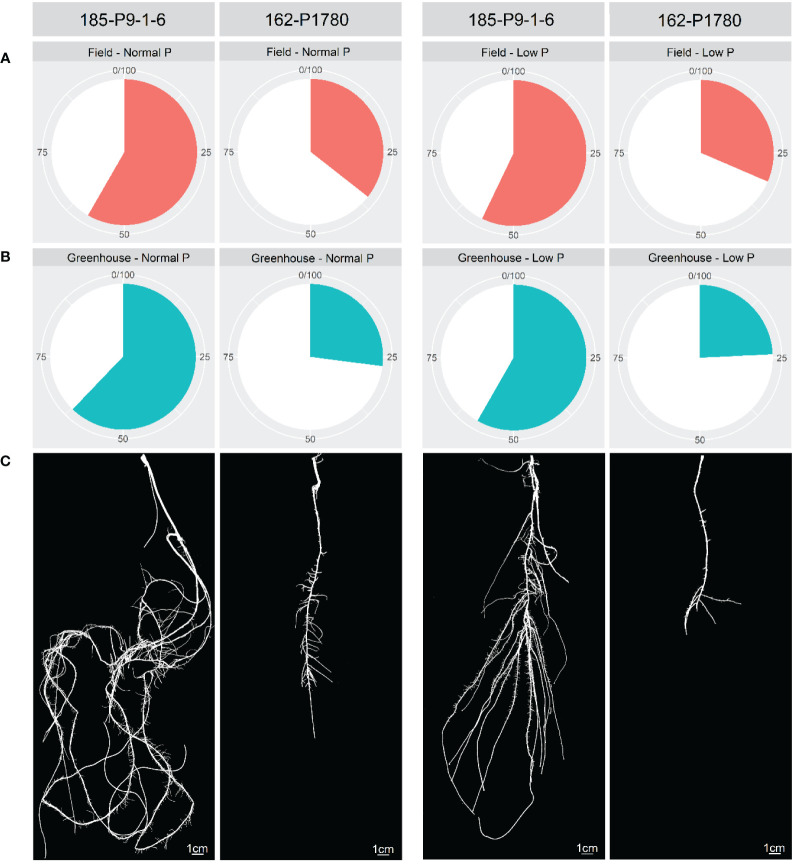
Percentage of favorable alleles of two contrasting maize inbred lines for P-efficiency in the field **(A)** and greenhouse **(B)** under low and normal P conditions. The root system of the 185-P9-1-6 and 162-P1788 inbred lines under low and normal P conditions **(C)**.

Considering only the favorable alleles related to PUE and its components, a gradual increase in these traits was observed by pyramiding these favorable alleles (**Figure 4**). Popcorn lines 17-P9-1-6 and 162-P1780 had the highest and lowest number of favorable alleles considering all traits, respectively (**Figure 5**). The 17-P9-1-6 line, considered P-efficient, presented 58.33% (field in normal P), 57.14% (field in low P), 62.16% (greenhouse in normal P), and 58.27% (greenhouse under low P) of the favorable alleles. On the other hand, line 162P1780, considered P-inefficient, presented only 32.18% (field in normal P), 31.45% (field in low P), 27.02% (greenhouse in normal P), and 24.13% (greenhouse under low P) of the favorable alleles.

### Candidate genes

A total of 186 potential candidate genes distributed in all maize chromosomes were identified (**Supplementary File 1**). Among them, 45 have already been annotated as classical or known genes in the maize genome, 21 of which have functions related to transcription factor activity, while the others are mainly associated with transport and transferase activity. According to the GO annotation, the primary molecular functions of the candidate genes included transcription factor, transferase, hydrolase, catalytic binding, ATP binding, DNA binding, and nucleotide-binding activity. In addition, several molecular functions directly related to P molecules were also identified, such as activities of phosphotransferase, phosphatase, pyrophosphatase, calcium-dependent phospholipid binding, all-trans-nonaprenyl-diphosphate synthase, glucose-6-phosphate isomerase, phosphorelay sensor kinase, phosphorylative mechanism, phospholipid binding, and phosphoenolpyruvate carboxylase.

## Discussion

### Genotype-environment interaction and genetic parameters

Genotype–season–P level interaction was observed in most of the traits evaluated in the field, indicating that the differential behavior of genotypes depends on the combination of P levels and seasons studied. Additionally, there were more significant interactions between genotype–season compared to genotype–P level interactions, suggesting that season strongly influences the evaluated traits. In the greenhouse trials, there was a significant genotype-P level interaction for all characteristics, indicating that the differential behavior of genotypes also depends on the P level in early evaluations. The genotype–P levels interaction in maize has already been reported in several greenhouse and field studies, which confirms that studies related to PUE and its components should be carried out under specific P conditions (Xu et al., 2018; Sahito et al., 2020; Li et al., 2021).

The negative influence of low P on the characteristics evaluated in the greenhouse and field was already expected since P is an essential element for plant development, constituting key cell molecules such as ATP, nucleic acids, and phospholipids, in addition to acting in the central regulation of many metabolisms, including energy transfer, protein activation, and carbon and amino acids metabolic processes (Wang et al., 2021). In contrast, the lines showed higher PUE, PutE, and PUpE under conditions of low P availability. In general, plants present higher PUE under low P since, under high phosphate fertilization, part of this element is lost in erosive processes and/or made unavailable by adsorption and immobilization processes in the soil (Alewell et al., 2020).

Although PUE-related traits are complex and strongly influenced by the environment, the heritability and selective accuracy estimates obtained in the present study fell into moderate to high, indicating favorable conditions for improvement aimed at increasing PUE. Heritability is the central parameter of any breeding program, used to estimate selection response and explain the proportion of phenotypic variation due to genetics (Hallauer et al., 2010). In the present study, heritability estimates were higher under normal P than low P conditions. In general, stress conditions tend to reduce heritability estimates, mainly due to the increased environmental influence on plant phenotype (Vats, 2018). Xu et al. (2018), evaluating 11 PUE-associated traits in maize, also observed reductions in heritability estimates under low P compared to normal P conditions.

### Correlation between traits

The existence of correlations among traits evaluated in the greenhouse and field trials would enable the early selection of genotypes with higher PUE. Unfortunately, however, no relevant correlations were observed between the traits evaluated in both trials, indicating that early selection in hydroponic systems may not be efficient in selecting plants with higher PUE under field conditions. Similar results were reported in other studies, suggesting that different genes and morphophysiological mechanisms act at different stages of plant development (Dissanayaka et al., 2018; Wang et al., 2019a). In fact, experiments in hydroponic systems or pots using nutrient solutions may present conditions that are very different from real field conditions since the environmental influence is much greater in the field than under greenhouse-controlled conditions (Araus et al., 2018).

P is relatively immobile in the soil, and its availability is greater at surface soil horizons, decreasing substantially at deeper soil horizons (Menezes-Blackburn et al., 2018). Thus, several studies have reported the importance of the maize root architecture system for higher PUE under field and greenhouse conditions (Jia et al., 2018; Klamer et al., 2019). The present study corroborated these results, showing a high correlation between the root-system-related traits (RB, RSA, RV, NR, and RL) with PUE in both P conditions under a hydroponic system. The indirect selection of PUE through root system traits is highly relevant in breeding programs, representing a faster and money-saving alternative, as it does not require laboratory analysis for P quantification. Further, high-yield phenotyping methods are already well established to characterize the root system of plants (Tracy et al., 2020).

### Linkage disequilibrium

Linkage disequilibrium (LD) is the non-random association of alleles at two or more loci and plays a central role in association mapping analyses (Romay et al., 2013). In the present study, we observed a rapid LD decay. In addition, we also report differences in LD decay in relation to chromosomes and correction methods assessed, with LD decay being mainly affected by population structure. Several studies have reported rapid LD decay in maize, ranging primarily from 0.1 to 10 kb (Yan et al., 2009; Romay et al., 2013; Bennetzen et al., 2018; Coan et al., 2018). In addition to the different approaches to estimating LD and LD decay, LD estimates may vary depending on the genomic region and germplasm evaluated (Vos et al., 2017). In maize, tropical and subtropical germplasms have faster LD decay when compared to temperate germplasms, as they have greater genetic diversity and the presence of rare alleles (Yan et al., 2009; Romay et al., 2013).

### Multi-locus GWAS methods

Multi-locus GWAS methods have been recently used to investigate the genetic basis of essential traits in several agricultural crops, such as maize (Lu et al., 2021), rice (Cui et al., 2018), bread wheat (Yang et al., 2020), soybean (Zhang et al., 2018), barley (Hu et al., 2018), and upland cotton (Su et al., 2018). In the present study, five different multi-locus GWAS methods were assessed. Among them, the FASTmrMLM and pLARmEB were the most efficient methods, as they detected more than 94% of the reliable QTNs in the present study, that is, QTNs co-detected by three or more methods. Although these methods present a combined two-step approach, the quantitative and qualitative differences in the identified QTNs are due to the different algorithms used in each method (Zhang et al., 2020). Thus, Zhang et al. (2019) suggested using combined results between the different methods of multi-locus GWAS to obtain more accurate results.

### QTNs identified

The present study identified a large number of small effects QTNs, confirming the complex and quantitative nature of PUE in maize. Using small-effect QTNs associated with traits of interest represents a smart strategy in genomic selection (GS) approaches since the use of these QTNs alone can replace the need for high-density genotyping through random SNPs, thus reducing genotyping costs (Lan et al., 2020). Furthermore, GS models using only SNPs known to be associated with traits of interest showed greater prediction accuracy, as they had lower background noise (Ali et al., 2020). In addition to their use in GS, these QTNs may be promising for application in breeding programs aimed at pyramiding favorable alleles through marker-assisted selection (MAS) (Boopathi, 2020).

In a QTLome study for low P tolerance in maize under limiting phosphorous conditions, Zhang et al. (2014) identified 23 meta-QTLs, that is, genomic regions more likely to harbor candidate genes responsible for traits related to tolerance to low P and/or PUE. Among the 306 QTNs discovered in the present study, 20 are located within the ranges of 11 meta-QTLs. In another analysis of meta-QTLs, Guo et al. (2018) identified 53 meta-QTLs associated with the maize root architecture system in the presence and absence of abiotic stresses (water deficit, high temperature, and P and N deficiency). Among the 54 QTNs found in the present study to be associated with root traits, 10 are located within the ranges of eight meta-QTLs. Thereby, the location of QTNs in genomic regions known to be associated with traits and characteristics of interest reinforces the significance and relevance of the QTNs pinpointed in the present study.

### Candidate genes

Among the 186 candidate genes detected in the present study, 45 have already been annotated as classical or known genes in maize. For instance, the *GRMZM2G083841* gene (associated with PUpE in LP_G) codes for a phosphoenolpyruvate carboxylase, catalyzing the carboxylation of phosphoenolpyruvate to produce oxaloacetate and Pi, performing the primary fixation of atmospheric CO_2_ in C4 plants (Nimmo, 2003). The *GRMZM2G140614* gene (associated with PUE in NP_C) encodes a glucose-6-phosphate isomerase, an essential enzyme in the oxidative pathway of pentose phosphate responsible for producing energy-rich cofactors and generation of carbon skeletons required for biosynthetic reactions (Yu et al., 2000). Interestingly, *GRMZM2G307119* (associated with RV in NP_G) was initially related to the formation of spikelet meristems (Chuck and Hake, 2005). However, Jansen et al. (2013) revealed that this gene plays a fundamental role in developing lateral roots in maize. In turn, the *GRMZM2G177792* gene (associated with PG in LP_C) has a peroxidase activity and has been associated with resistance to biotic stresses (Santiago et al., 2016; Musungu et al., 2020). In addition to these genes, three main mechanisms and pathways were discovered:

1. *Transcription regulator activity*. Transcription factors regulate gene expression. Previous studies have reported that many of these factors trigger P stress response and/or PUE in maize (Calderon-Vazquez et al., 2008; Xu et al., 2018). Of the 45 classical or known genes identified in the present study, 21 (~46%) are classified as transcription factors. The *GRMZM2G317160* gene (associated with PS in LP_19) is a member of the *AP2-EREBP* family of transcription factors and plays a significant role in abiotic stress response (Du et al., 2014). The *GRMZM2G479760* gene (associated with HI and PUE in NP_C) belongs to the *basic leucine zipper* (bZIP) family. It regulates several phenomena during maize growth and development and participates in responses to abiotic stresses and hormonal signaling (Cao et al., 2019). *GRMZM5G808366* (associated with HI in LP_18) belongs to the auxin response factor (ARF) gene family and has an elevated expression level under P stress conditions (Pei et al., 2013). In turn, the *GRMZM2G073823* gene (associated with RB in NP_G) is already known as an important gene during maize root growth and development (Jiang et al., 2012; Li et al., 2019b).
2. *Transporter activity*. Several specific and non-specific nutrient transporters have been reported in response to P deficiency stress (Shabala et al., 2016; Xu et al., 2018; Wang et al., 2021). *GRMZM2G161459* (associated with PUtE at NP_18) encodes the transport peptide PTR2 that transports a broad spectrum of dipeptides and is involved in several pathways (Li et al., 2016a). *GRMZM2G064467* (associated with PUE in LP_C) is involved in magnesium transmembrane transporter activity and responds to abiotic stresses (Li et al., 2016b). *GRMZM2G455557* (associated with PHI in NP_C) encodes a plasma membrane H^+^-ATPase, creating electrochemical gradients for soil nutrient uptake by roots and is involved in additional solute xylem loading (Falhof et al., 2016). This gene has already been associated with N assimilation in maize (Plett et al., 2016), while other genes related to plasma membrane H^+^-ATPase have been identified in response to P deficiency (Yuan et al., 2017; Stein et al., 2019).
3. *Transferase activity*. Transferases are enzymes that transfer functional groups from one molecule to another, catalyzing several reactions involving nutrient absorption, translocation, and storage. They also respond to abiotic stresses. Among the genes identified in this study, *GRMZM2G104511* (associated with PUE in LP_18) encodes a protein from the *O-fucosyltransferase* family, physically interacting with proteins involved in cell division and responses to stress and hormones (Jia et al., 2013). This gene was differentially expressed in maize under water deficit (Zheng et al., 2020) and P conditions (Du et al., 2016). The *GRMZM5G851405* gene (associated with PHI in NP_18) encodes a histone acetyltransferase whose function is to catalyze the acetylation of central histones by adding an acetyl group to the lysine residue in the terminal tail of histones. The regulation of histone acetyltransferases has already been associated with the phosphate starvation response in *Arabidopsis thaliana* (Wang et al., 2019b). As for *GRMZM2G033767* (associated with PUtE in NP_G), it codes for a glycerol-3-phosphate acyltransferase, an enzyme that catalyzes an acyl group from an acyl donor to the sn-1 position of glycerol 3-phosphate and has great importance in regulating lipid biosynthesis (Murata and Tasaka, 1997). Finally, the *GRMZM2G141810* gene (associated with PS in LP_19) encodes a tryptophan aminotransferase and is importantly related to nutrient storage functions in maize endosperm (Bernardi et al., 2012; Zhan et al., 2018).

## Conclusion

Wide genetic variability was observed for PUE, and we confirmed its complex nature. On the other hand, we did not verify relevant correlations between traits evaluated in the greenhouse and field, indicating that early screening may not be efficient in selecting genotypes with higher PUE under field conditions. A total of 306 QTNs were associated with the 24 evaluated traits in the present study using different multi-locus GWAS models. From these QTNs, 186 potential candidates were identified, mainly involved with transcription regulators, transporters, and transference activities. Our study provides new insights into PUE genetic architecture and may serve as a basis for further functional investigation. In addition, the QTNs detected in this study can be used for pyramiding favorable alleles to develop maize varieties with higher PUE and, consequently, less dependent on phosphate fertilization.

## Data Availability

The data presented in the study are deposited in the at European Variation Archive (EVA) repository, accession number PRJEB72805.

## References

[B1] Alewell C. Ringeval B. Ballabio C. Robinson D. A. Panagos P. Borrelli P. (2020). Global phosphorus shortage will be aggravated by soil erosion. Nat. Commun. 11, 4546. doi: 10.1038/s41467-020-18326-7 32917863 PMC7486398

[B2] Ali M. Zhang Y. Rasheed A. Wang J. Zhang L. (2020). Genomic prediction for grain yield and yield-related traits in chinese winter wheat. Int. J. Mol. Sci. 21, 1342. doi: 10.3390/ijms21041342 32079240 PMC7073225

[B3] Araus J. L. Kefauver S. C. Zaman-Allah M. Olsen M. S. Cairns J. E. (2018). Translating high-throughput phenotyping into genetic gain. Trends Plant Sci. 23, 451–466. doi: 10.1016/j.tplants.2018.02.001 29555431 PMC5931794

[B4] Bennetzen J. Flint-Garcia S. Hirsch C. Tuberosa R. (Eds.) (2018). The maize genome (New York: Springer International Publishing). doi: 10.1007/978-3-319-97427-9

[B5] Bernardi J. Lanubile A. Li Q. B. Kumar D. Kladnik A. Cook S. D.. (2012). Impaired auxin biosynthesis in the defective endosperm18 mutant is due to mutational loss of expression in the ZmYuc1 gene encoding endosperm-specific YUCCA1 protein in maize. Plant Physiol. 160, 1318–1328. doi: 10.1104/pp.112.204743 22961134 PMC3490580

[B6] Bernardino K. C. Pastina M. M. Menezes C. B. Souza S. M. Maciel L. S. Jr Carvalho G.. (2019). The genetic architecture of phosphorus efficiency in sorghum involves pleiotropic QTL for root morphology and grain yield under low phosphorus availability in the soil. BMC Plant Biol. 19, 87. doi: 10.1186/s12870-019-1689-y 30819116 PMC6394046

[B7] Bindraban P. S. Dimkpa C. O. Pandey R. (2020). Exploring phosphorus fertilizers and fertilization strategies for improved human and environmental health. Biol. Fertil Soils 56, 299–317. doi: 10.1007/s00374-019-01430-2

[B8] Boopathi N. M. (2020). “Marker-assisted selection (MAS),” in Genetic mapping and marker assisted selection (Springer, Singapore), 343–388. doi: 10.1007/978-981-15-2949-8_9

[B9] Bradbury P. J. Zhang Z. Kroon D. E. Casstevens T. M. Ramdoss Y. Buckler E. S. (2007). TASSEL: software for association mapping of complex traits in diverse samples. Bioinformatics 23, 2633–2635. doi: 10.1093/bioinformatics/btm308 17586829

[B10] Browning B. L. Zhou Y. Browning. S. R. (2018). A one-penny imputed genome from next-generation reference panels. Am. J. Hum. Genet. 103, 338–348. doi: 10.1016/j.ajhg.2018.07.015 30100085 PMC6128308

[B11] Byerlee D. (2020). The globalization of hybrid maize 1921–70. J. Glob Hist 15, 101–122. doi: 10.1017/S1740022819000354

[B12] Calderon-Vazquez C. Ibarra-Laclette E. Caballero-Perez J. Herrera-Estrella L. (2008). Transcript profiling of Zea mays roots reveals gene responses to phosphate deficiency at the plant-and species-specific levels. J. Exp. Bot. 59, 2479–2497. doi: 10.1093/jxb/ern115 18503042

[B13] Cao L. Lu X. Zhang P. Wang G. Wei L. Wang T. (2019). Systematic analysis of differentially expressed maize ZmbZIP genes between drought and rewatering transcriptome reveals bZIP family members involved in abiotic stress responses. Int. J. Mol. Sci. 20, 4103. doi: 10.3390/ijms20174103 31443483 PMC6747360

[B14] Chuck G. Hake S. (2005). Regulation of developmental transitions. Curr. Opin. Plant Biol. 8, 67–70. doi: 10.1016/j.pbi.2004.11.002 15653402

[B15] Coan M. M. Senhorinho H. J. Pinto R. J. Scapim C. A. Tessmann D. J. Williams W. P.. (2018). Genome-wide association study of resistance to ear rot by Fusarium verticillioides in a tropical field maize and popcorn core collection. Crop Sci. 58, 564–578. doi: 10.2135/cropsci2017.05.0322

[B16] Cortes L. T. Zhang Z. Yu J. (2021). Status and prospects of genome-wide association studies in plants. Plant Genome 14, e20077. doi: 10.1002/tpg2.20077 33442955 PMC12806871

[B17] Cui Y. Zhang F. Zhou Y. (2018). The application of multi-locus GWAS for the detection of salt-tolerance loci in rice. Front. Plant Sci. 9. doi: 10.3389/fpls.2018.01464 PMC6180169 30337936

[B18] Danecek P. Auton A. Abecasis G. Albers C. A. Banks E. DePristo M. A.. (2011). The variant call format and VCFtools. Bioinformatics 27, 2156–2158. doi: 10.1093/bioinformatics/btr330 21653522 PMC3137218

[B19] Dissanayaka D. M. S. B. Ghahremani M. Siebers M. Wasaki J. Plaxton W. C. (2021). Recent insights into the metabolic adaptations of phosphorus-deprived plants. J. Exp. Bot. 72, 199–223. doi: 10.1093/jxb/eraa482 33211873

[B20] Dissanayaka D. M. S. B. Plaxton W. C. Lambers H. Siebers M. Marambe B. Wasaki J. (2018). Molecular mechanisms underpinning phosphorus-use efficiency in rice. Plant Cell Environ. 41, 1483–1496. doi: 10.1111/pce.13191 29520969

[B21] Du H. Huang M. Zhang Z. Cheng S. (2014). Genome-wide analysis of the AP2/ERF gene family in maize waterlogging stress response. Euphytica 198, 115–126. doi: 10.1007/s10681-014-1088-2

[B22] Du Q. Wang K. Xu C. Zou C. Xie C. Xu Y.. (2016). Strand-specific RNA-Seq transcriptome analysis of genotypes with and without low-phosphorus tolerance provides novel insights into phosphorus-use efficiency in maize. BMC Plant Biol. 16, 222. doi: 10.1186/s12870-016-0903-4 27724863 PMC5057381

[B23] Earl D. A. (2012). STRUCTURE HARVESTER: a website and program for visualizing STRUCTURE output and implementing the Evanno method. Conserv. Genet. Resour 4, 359–361. doi: 10.1007/s12686-011-9548-7

[B24] Elshire R. J. Glaubitz J. C. Sun Q. Poland J. A. Kawamoto K. Buckler E. S.. (2011). A robust, simple genotyping-by-sequencing (GBS) approach for high diversity species. PloS One 6, e19379. doi: 10.1371/journal.pone.0019379 21573248 PMC3087801

[B25] Endelman J. B. Jannink J. L. (2012). Shrinkage estimation of the realized relationship matrix. G3 (Bethesda) 2, 1405–1413. doi: 10.1534/g3.112.004259 23173092 PMC3484671

[B26] Evanno G. Regnaut S. Goudet J. (2005). Detecting the number of clusters of individuals using the software STRUCTURE: a simulation study. Mol. Ecol. 14, 2611–2620. doi: 10.1111/j.1365-294X.2005.02553.x 15969739

[B27] Falhof J. Pedersen J. T. Fuglsang A. T. Palmgren M. (2016). Plasma membrane H+-ATPase regulation in the center of plant physiology. Mol. Plant 9, 323–337. doi: 10.1016/j.molp.2015.11.002 26584714

[B28] Galkovskyi T. Mileyko Y. Bucksch A. Moore B. Symonova O. Price C. A.. (2012). GiA Roots: software for the high throughput analysis of plant root system architecture. BMC Plant Biol. 12, 116. doi: 10.1186/1471-2229-12-116 22834569 PMC3444351

[B29] Glaser B. Lehr V. I. (2019). Biochar effects on phosphorus availability in agricultural soils: A meta-analysis. Sci. Rep. 9, 1–9. doi: 10.1038/s41598-019-45693-z 31249335 PMC6597700

[B30] Glaubitz J. C. Casstevens T. M. Lu F. Harriman J. Elshire R. J. Sun Q.. (2014). TASSEL-GBS: a high capacity genotyping by sequencing analysis pipeline. PloS One 9, e90346. doi: 10.1371/journal.pone.0090346 24587335 PMC3938676

[B31] Gu R. Chen F. Long L. Cai H. Liu Z. Yang J.. (2016). Enhancing phosphorus uptake efficieny through QTL-based selection for root system architecture in maize. J. Genet. Genomics 43, 663–672. doi: 10.1016/j.jgg.2016.11.002 27889500

[B32] Guo J. Chen L. Li Y. Shi Y. Song Y. Zhang D.. (2018). Meta-QTL analysis and identification of candidate genes related to root traits in maize. Euphytica 214, 223. doi: 10.1007/s10681-018-2283-3

[B33] Hallauer A. R. Carena M. J. Miranda Filho J. D. (2010). Quantitative genetics in maize breeding (vol. 6) (New York: Springer Science & Business Media). doi: 10.1007/978-1-4419-0766-0

[B34] Hill W. G. Weir B. S. (1988). Variances and covariances of squared linkage disequilibria in finite populations. Theor. Popul Biol. 33, 54–78. doi: 10.1016/0040-5809(88)90004-4 3376052

[B35] Hu X. Zuo J. Wang J. Liu L. Sun G. Li C.. (2018). Multi-locus genome-wide association studies for 14 main agronomic traits in barley. Front. Plant Sci. 9. doi: 10.3389/fpls.2018.01683 PMC6257129 30524459

[B36] Iqbal S. Akhtar J. Naz T. Riaz U. Hussain S. Mazhar Z.. (2020). Root morphological adjustments of crops to improve nutrient use efficiency in limited environments. Commun. Soil Sci. Plant Anal. 51, 2452–2465. doi: 10.1080/00103624.2020.1836199

[B37] Jansen L. Hollunder J. Roberts I. Forestan C. Fonteyne P. Van Quickenborne C.. (2013). Comparative transcriptomics as a tool for the identification of root branching genes in maize. Plant Biotechnol. J. 11, 1092–1102. doi: 10.1111/pbi.12104 23941360

[B38] Jia F. Wu B. Li H. Huang J. Zheng C. (2013). Genome-wide identification and characterisation of F-box family in maize. Mol. Genet. Genom 288, 559–577. doi: 10.1007/s00438-013-0769-1 23928825

[B39] Jia X. Liu P. Lynch J. P. (2018). Greater lateral root branching density in maize improves phosphorus acquisition from low phosphorus soil. J. Exp. Bot. 69, 4961–4970. doi: 10.1093/jxb/ery252 30295904 PMC6137997

[B40] Jiang Y. Zeng B. Zhao H. Zhang M. Xie S. Lai J. (2012). Genome-wide transcription factor gene prediction and their expressional tissue-specificities in maize. J. Integr. Plant Biol. 54, 616–630. doi: 10.1111/j.1744-7909.2012.01149.x 22862992

[B41] Klamer F. Vogel F. Li X. Bremer H. Neumann G. Neuhäuser B.. (2019). Estimating the importance of maize root hairs in low phosphorus conditions and under drought. Ann. Bot. 124, 961–968. doi: 10.1093/aob/mcz011 30759179 PMC6881218

[B42] Lan S. Zheng C. Hauck K. McCausland M. Duguid S. D. Booker H. M.. (2020). Genomic prediction accuracy of seven breeding selection traits improved by QTL identification in flax. Int. J. Mol. Sci. 21, 1577. doi: 10.3390/ijms21051577 32106624 PMC7084455

[B43] Li D. Chen Z. Wang M. Leiser W. L. Weiß T. M. Zhao Z.. (2021). Dissecting the phenotypic response of maize to low phosphorus soils by field screening of a large diversity panel. Euphytica 217, 1–12. doi: 10.1007/s10681-020-02727-2

[B44] Li D. Wang M. Kuang X. Liu W. (2019a). Genetic study and molecular breeding for high phosphorus use efficiency in maize. Front. Agric. Sci. Eng. 6, 366–379. doi: 10.15302/J-FASE-2019278

[B45] Li H. Du H. Huang K. Chen X. Liu T. Gao S.. (2016b). Identification, and functional and expression analyses of the CorA/MRS2/MGT-type magnesium transporter family in maize. Plant Cell Physiol. 57, 1153–1168. doi: 10.1093/pcp/pcw064 27084594

[B46] Li X. Zhou Z. Ding J. Wu Y. Zhou B. Wang R.. (2016a). Combined linkage and association mapping reveals QTL and candidate genes for plant and ear height in maize. Front. Plant Sci. 7. doi: 10.3389/fpls.2016.00833 PMC4908132 27379126

[B47] Li Y. Liu X. Chen R. Tian J. Fan Y. Zhou X. (2019b). Genome-scale mining of root-preferential genes from maize and characterization of their promoter activity. BMC Plant Biol. 19, 1–12. doi: 10.1186/s12870-019-2198-8 31878892 PMC6933907

[B48] Lu X. Wang J. Wang Y. Wen W. Zhang Y. Du J.. (2021). Genome-wide association study of maize aboveground dry matter accumulation at seedling stage. Front. Genet. 11. doi: 10.3389/fgene.2020.571236 PMC7838602 33519889

[B49] Magnavaca R. Gardner C. O. Clark R. B. (1987). “Evaluation of inbred maize lines for aluminum tolerance in nutrient solution,” in Genetic aspects of plant mineral nutrition (Springer, Dordrecht), 255–265). doi: 10.1007/978-94-009-3581-5_23

[B50] Malavolta E. Vitti G. C. de Oliveira S. A. (1989). Evaluation of the nutritional state of plants: principles and applications. (Piracicaba: Associação Brasileira para Pesquisa de Potássio e Fósforo).

[B51] Mangin B. Siberchicot A. Nicolas S. Doligez A. This P. Cierco-Ayrolles C. (2012). Novel measures of linkage disequilibrium that correct the bias due to population structure and relatedness. Heredity 108, 285–291. doi: 10.1038/hdy.2011.73 21878986 PMC3282397

[B52] Meirelles W. F. Parentoni S. N. Guimarães L. J. M. Guimarães P. E. O. Pacheco C. A. P. de Oliveira A. C.. (2016). Diallel anlysis of maize lines as to their phosphorus responsiveness and use efficiency. Pesq Agropec Bras. 51, 224–232. doi: 10.1590/S0100-204X2016000300004

[B53] Mendes F. F. Guimarães L. J. M. Souza J. C. Guimarães P. E. O. Magalhaes J. V. Garcia A. A. F.. (2014). Genetic architecture of phosphorus use efficiency in tropical maize cultivated in a low-P soil. Crop Sci. 54, 1530–1538. doi: 10.2135/cropsci2013.11.0755

[B54] Menezes-Blackburn D. Giles C. Darch T. George T. S. Blackwell M. Stutter M.. (2018). Opportunities for mobilizing recalcitrant phosphorus from agricultural soils: a review. Plant Soil 427, 5–16. doi: 10.1007/s11104-017-3362-2 30996482 PMC6438637

[B55] Moll R. H. Kamprath E. J. Jackson W. A. (1982). Analysis and interpretation of factors which contribute to efficiency of nitrogen utilization. Agron. J. 74, 562–564. doi: 10.2134/agronj1982.00021962007400030037x

[B56] Murata N. Tasaka Y. (1997). Glycerol-3-phosphate acyltransferase in plants. Biochim. Biophys. Acta Lipids Lipid Metab. 1348, 10–16. doi: 10.1016/S0005-2760(97)00115-X 9370311

[B57] Musungu B. Bhatnagar D. Quiniou S. Brown R. L. Payne G. A. O’Brian G.. (2020). Use of Dual RNA-seq for Systems Biology Analysis of Zea mays and Aspergillus flavus interaction. Front. Microbiol. 11. doi: 10.3389/fmicb.2020.00853 PMC7285840 32582038

[B58] Nimmo H. G. (2003). Control of the phosphorylation of phosphoenolpyruvate carboxylase in higher plants. Arch. Biochem. Biophys. 414, 189–196. doi: 10.1016/S0003-9861(03)00115-2 12781770

[B59] Parentoni S. N. Souza Junior C. L. Alves V. M. C. Gama E. E. G. Coelho A. M.. (2010). Inheritance and breeding strategies for phosphorus efficiency in tropical maize (*Zea mays* L.). Maydica 55, 1–15.

[B60] Pavinato P. S. Cherubin M. R. Soltangheisi A. Rocha G. C. Chadwick D. R. Jones D. L. (2020). Revealing soil legacy phosphorus to promote sustainable agriculture in Brazil. Sci. Rep. 10, 1–11. doi: 10.1038/s41598-020-72302-1 32985529 PMC7522976

[B61] Pei L. Jin Z. Li K. Yin H. Wang J. Yang A. (2013). Identification and comparative analysis of low phosphate tolerance-associated microRNAs in two maize genotypes. Plant Physiol. Biochem. 70, 221–234. doi: 10.1016/j.plaphy.2013.05.043 23792878

[B62] Plett D. Holtham L. Baumann U. Kalashyan E. Francis K. Enju A.. (2016). Nitrogen assimilation system in maize is regulated by developmental and tissue-specific mechanisms. Plant Mol. Biol. 92, 293–312. doi: 10.1007/s11103-016-0512-5 27511191

[B63] Pradhan S. Pokhrel M. R. (2013). Spectrophotometric determination of phosphate in sugarcane juice, fertilizer, detergent and water samples by molybdenum blue method. Sci. World 11, 58–62.

[B64] Pritchard J. K. Stephens M. Donnelly P. (2000). Inference of population structure using multilocus genotype data. Genetics 155, 945–959. doi: 10.1093/genetics/155.2.945 10835412 PMC1461096

[B65] Resende M. D. V. D. (2016). Software Selegen-REML/BLUP: a useful tool for plant breeding. Crop Breed Appl. Biotechnol. 16, 330–339. doi: 10.1590/1984-70332016v16n4a49

[B66] Rodrigues M. Withers P. J. A. Soltangheisi A. Vargas V. Holzschuh M. Pavinato P. S. (2021). Tillage systems and cover crops affecting soil phosphorus bioavailability in Brazilian Cerrado Oxisols. Soil Tillage Res. 205, 104770. doi: 10.1016/j.still.2020.104770

[B67] Romay M. C. Millard M. J. Glaubitz J. C. Peiffer J. A. Swarts K. L. Casstevens T. M.. (2013). Comprehensive genotyping of the USA national maize inbred seed bank. Genome Biol. 14, 1–18. doi: 10.1186/gb-2013-14-6-r55 PMC3707059 23759205

[B68] Sahito J. H. Zheng F. Tang H. He X. Luo B. Zhang X.. (2020). Identification, association, and expression analysis of ZmNAC134 gene response to phosphorus deficiency tolerance traits in maize at seedling stage. Euphytica 216, 100. doi: 10.1007/s10681-020-02634-6

[B69] Santiago R. Malvar R. A. Barros-Rios J. Samayoa L. F. Butrón A. (2016). Hydroxycinnamate synthesis and association with Mediterranean corn borer resistance. J. Agric. Food Chem. 64, 539–551. doi: 10.1021/acs.jafc.5b04862 26690311

[B70] Seleiman M. F. Almutairi K. F. Alotaibi M. Shami A. Alhammad B. A. Battaglia M. L. (2021). Nano-fertilization as an emerging fertilization technique: why can modern agriculture benefit from its use? Plants 10, 2. doi: 10.3390/plants10010002 PMC7822031 33375026

[B71] Shabala S. Bose J. Fuglsang A. T. Pottosin I. (2016). On a quest for stress tolerance genes: membrane transporters in sensing and adapting to hostile soils. J. Exp. Bot. 67, 1015–1031. doi: 10.1093/jxb/erv465 26507891

[B72] Stein S. Faust F. Jung S. Schubert S. (2019). Expression of plasma membrane H+-ATPase in cluster roots of white lupin under phosphorus deficiency. J. Plant Nutr. Soil Sci. 182, 867–870. doi: 10.1002/jpln.201900124

[B73] Su J. Ma Q. Li M. Hao F. Wang C. (2018). Multi-locus genome-wide association studies of fiber-quality related traits in Chinese early-maturity upland cotton. Front. Plant Sci. 9. doi: 10.3389/fpls.2018.01169 PMC6107031 30166989

[B74] Tamba C. L. Ni Y. L. Zhang Y. M. (2017). Iterative sure independence screening EM-Bayesian LASSO algorithm for multi-locus genome-wide association studies. PloS Comput. Biol. 13, e1005357. doi: 10.1371/journal.pcbi.1005357 28141824 PMC5308866

[B75] Tamba C. L. Zhang Y. M. (2018). A fast mrMLM algorithm for multi-locus genome-wide association studies. bioRxiv, 341784. doi: 10.1101/341784

[B76] Tracy S. R. Nagel K. A. Postma J. A. Fassbender H. Wasson A. Watt M. (2020). Crop improvement from phenotyping roots: highlights reveal expanding opportunities. Trends Plant Sci. 25, 105–118. doi: 10.1016/j.tplants.2019.10.015 31806535

[B77] Vats S. (Ed.) (2018). Biotic and abiotic stress tolerance in plants (Singapore: Springer). doi: 10.1007/978-981-10-9029-5

[B78] Vos P. G. Paulo M. J. Voorrips R. E. Visser R. G. van Eck H. J. van Eeuwijk F. A. (2017). Evaluation of LD decay and various LD-decay estimators in simulated and SNP-array data of tetraploid potato. Theor. Appl. Genet. 130, 123–135. doi: 10.1007/s00122-016-2798-8 27699464 PMC5214954

[B79] Wang Q. Yuan Y. Liao Z. Jiang Y. Wang Q. Yuan Y. Liao Z. Jiang Y. Wang Q. Zhang T.. (2019). Genome-wide association study of 13 traits in maize seedlings under low phosphorus stress. Plant Genome 12, 190039. doi: 10.3835/plantgenome2019.06.0039 PMC12810056 33016582

[B80] Wang S. B. Feng J. Y. Ren W. L. Huang B. Zhou L. Wen Y. J.. (2016). Improving power and accuracy of genome-wide association studies via a multi-locus mixed linear model methodology. Sci. Rep. 6, 1–10. doi: 10.1038/srep19444 26787347 PMC4726296

[B81] Wang T. Xing J. Liu Z. Zheng M. Yao Y. Hu Z.. (2019b). Histone acetyltransferase GCN5-mediated regulation of long non-coding RNA At4 contributes to phosphate starvation response in Arabidopsis. J. Exp. Bot. 70, 6337–6348. doi: 10.1093/jxb/erz359 31401648 PMC6859718

[B82] Wang W. Ding G. D. White P. J. Wang X. H. Jin K. M. Xu F. S.. (2019a). Mapping and cloning of quantitative trait loci for phosphorus efficiency in crops: opportunities and challenges. Plant Soil 439, 91–112. doi: 10.1007/s11104-018-3706-6

[B83] Wang Y. Lambers H. (2020). Root-released organic anions in response to low phosphorus availability: recent progress, challenges and future perspectives. Plant Soil 447, 135–156. doi: 10.1007/s11104-019-03972-8

[B84] Wang Y. Chen Y. F. Wu W. H. (2021). Potassium and phosphorus transport and signaling in plants. J. Integr. Plant Biol. 63, 34–52. doi: 10.1111/jipb.13053 33325114

[B85] Wen Y. J. Zhang H. Ni Y. L. Huang B. Zhang J. Feng J. Y.. (2018). Methodological implementation of mixed linear models in multi-locus genome-wide association studies. Brief Bioinform. 19, 700–712. doi: 10.1093/bib/bbw145 28158525 PMC6054291

[B86] Withers P. J. Rodrigues M. Soltangheisi A. De Carvalho T. S. Guilherme L. R. Benites V. D. M.. (2018). Transitions to sustainable management of phosphorus in Brazilian agriculture. Sci. Rep. 8, 1–13. doi: 10.1038/s41598-018-20887-z 29416090 PMC5803245

[B87] Xu C. Zhang H. Sun J. Guo Z. Zou C. Li W. X.. (2018). Genome-wide association study dissects yield components associated with low-phosphorus stress tolerance in maize. Theor. Appl. Genet. 131, 1699–1714. doi: 10.1007/s00122-018-3108-4 29754325

[B88] Yan J. Shah T. Warburton M. L. Buckler E. S. McMullen M. D. Crouch J. (2009). Genetic characterization and linkage disequilibrium estimation of a global maize collection using SNP markers. PloS One 4, e8451. doi: 10.1371/journal.pone.0008451 20041112 PMC2795174

[B89] Yang Y. Chai Y. Zhang X. Lu S. Zhao Z. Wei D.. (2020). Multi-locus GWAS of quality traits in bread wheat: mining more candidate genes and possible regulatory network. Front. Plant Sci. 11. doi: 10.3389/fpls.2020.01091 PMC7411135 32849679

[B90] Yu T. S. Lue W. L. Wang S. M. Chen J. (2000). Mutation of Arabidopsis plastid phosphoglucose isomerase affects leaf starch synthesis and floral initiation. Plant Physiol. 123, 319–326. doi: 10.1104/pp.123.1.319 10806248 PMC59005

[B91] Yuan W. Zhang D. Song T. Xu F. Lin S. Xu W.. (2017). Arabidopsis plasma membrane H+-ATPase genes AHA2 and AHA7 have distinct and overlapping roles in the modulation of root tip H+ efflux in response to low-phosphorus stress. J. Exp. Bot. 68, 1731–1741. doi: 10.1093/jxb/erx040 28369625 PMC5441905

[B92] Zhan J. Li G. Ryu C. H. Ma C. Zhang S. Lloyd A.. (2018). Opaque-2 regulates a complex gene network associated with cell differentiation and storage functions of maize endosperm. Plant Cell 30, 2425–2446. doi: 10.1105/tpc.18.00392 30262552 PMC6241275

[B93] Zhang H. Uddin M. S. Zou C. Xie C. Xu Y. Li W. X. (2014). Meta-analysis and candidate gene mining of low-phosphorus tolerance in maize. J. Integr. Plant Biol. 56, 262–270. doi: 10.1111/jipb.12168 24433531

[B94] Zhang J. Feng J. Y. Ni Y. L. Wen Y. J. Niu Y. Tamba C. L.. (2017). pLARmEB: integration of least angle regression with empirical Bayes for multilocus genome-wide association studies. Heredity 118, 517–524. doi: 10.1038/hdy.2017.8 28295030 PMC5436030

[B95] Zhang K. Liu S. Li W. Liu S. Li X. Fang Y.. (2018). Identification of QTNs controlling seed protein content in soybean using multi-locus genome-wide association studies. Front. Plant Sci. 9. doi: 10.3389/fpls.2018.01690 PMC6258895 30519252

[B96] Zhang Y. M. Jia Z. Dunwell J. M. (2019). The applications of new multi-locus GWAS methodologies in the genetic dissection of complex traits. Front. Plant Sci. 10. doi: 10.3389/fpls.2019.00100 PMC6378272 30804969

[B97] Zhang Y. W. Tamba C. L. Wen Y. J. Li P. Ren W. L. Ni Y. L.. (2020). mrMLM v4. 0: An R platform for multi-locus genome-wide association studies. Genomics Proteomics Bioinf. 18, 481–487. doi: 10.1016/j.gpb.2020.06.006 PMC8242264 33346083

[B98] Zheng H. Yang Z. Wang W. Guo S. Li Z. Liu K.. (2020). Transcriptome analysis of maize inbred lines differing in drought tolerance provides novel insights into the molecular mechanisms of drought responses in roots. Plant Physiol. Biochem. 149, 11–26. doi: 10.1016/j.plaphy.2020.01.027 32035249

