# Supplementary material for: Multi-locus genome-wide association study for phosphorus use efficiency in a tropical maize germplasm
Source: Front Plant Sci. 2024 Aug 23;15:1366173. doi: 10.3389/fpls.2024.1366173 (PMC11380136; doi:10.3389/fpls.2024.1366173)
Supplement: Supplementary file 1 [file DataSheet1.pdf]

Multi-locus genome-wide association study for phosphorus use efficiency in a tropical maize germplasm

Douglas Mariani Zeffa[1], Luiz Perini Júnior[2], Rafael de Assis[3], Jéssica Delfini[2], Antoni Wallace Marcos[2], Alessandra Koltun[1], Viviane Yumi Baba[2], Leonel Vinícius Constantino[2], Alison Fernando Nogueira[2], Vania Moda-Cirino[4], Carlos Alberto Scapim[1] and Leandro Simões Azeredo Gonçalves[2*]

[1]Universidade Estadual de Maringá, Departamento de Agronomia, Maringá, Paraná, Brazil.

[2]Universidade Estadual de Londrina, Departamento de Agronomia, Londrina, Paraná, Brazil.

[3]Universidade Estadual de Londrina, Departamento de Biologia, Londrina, Paraná, Brazil

[4]Instituto de Desenvolvimento Rural do Paraná, Área de Melhoramento Genético e Propagação Vegetal, Londrina, Paraná, Brazil.

*Corresponding author: leandrosag@uel.br

Supplementary material

Supplementary Table 1 List of 132 maize inbred lines evaluated.

Inbred Line	Name	Inbred Line	Name
Field corn	102-PREMIUM-28H13.2	Popcorn	100-P7-2-3
Field corn	103-CML19	Popcorn	104-P1-3
Field corn	105-DKB747-45H17.5	Popcorn	106-ANGELA-L70
Field corn	108-FORT-85H6.2-242	Popcorn	107-GP11-1
Field corn	109-DKB747-29H17.3	Popcorn	117-GP14
Field corn	111-AG8080-8H3.2-6	Popcorn	119-VIcOSA-L77
Field corn	115-DKB747-37H17.2	Popcorn	121-ANGELA-L71
Field corn	11-DKB350-78H30.1	Popcorn	127-ANGELA-L66
Field corn	122-30F33-70H23.1	Popcorn	128-P3-1-2
Field corn	126-30F98-75H29.2	Popcorn	134-P9-5-3
Field corn	12-DKB747-50H17.6	Popcorn	141-P9-1-3
Field corn	131-CD303-90H4.3	Popcorn	144-P9-11-1
Field corn	136-TORK-53H20.2	Popcorn	145-BEIJAFLOR-L76

Field corn	137-POP203-56.1	Popcorn	147-P8-1-5-5
Field corn	138-POP102-166.5	Popcorn	148-P9-8-1
Field corn	139-A2560-62H23.2	Popcorn	14-P8-1-1
Field corn	140-POP101-195.2	Popcorn	152-P8-1-5-13
Field corn	142-FLASH-22H11.1	Popcorn	157-GP12
Field corn	149-TORK-55H20.3	Popcorn	15-P20
Field corn	151-FORT-84H6.1	Popcorn	161-P6-11
Field corn	153-CD303-91.H4.4	Popcorn	162-P1780
Field corn	155-DAS2C599-95H34.4	Popcorn	17-P1-9
Field corn	156-CML12	Popcorn	183-P9-2-3
Field corn	160-SPEED-81H33.1	Popcorn	185-P9-1-6
Field corn	163-POP102-91.2	Popcorn	18-P11-1
Field corn	164-30-11	Popcorn	195-BEIJAFLOR-L55
Field corn	165-POP101-197.1	Popcorn	19-P3-3T
Field corn	167-29-154	Popcorn	1-GP1
Field corn	16-FORT-87H6.4-248	Popcorn	200-URUG298-98-2
Field corn	170-DKB747-40H17.3	Popcorn	207-SAM274-2
Field corn	171-FORT-86H6.3	Popcorn	20-T1-P8-2
Field corn	174-A2560-176	Popcorn	26-P1-12
Field corn	175-A2560-164	Popcorn	27-GP13
Field corn	176-DKB747-41-101	Popcorn	28-P9-1
Field corn	177-DKB747-47-121	Popcorn	29-P7-2-4
Field corn	189-DKB440-73-H28.1	Popcorn	2-GP4
Field corn	190-Premium-29-h13.3	Popcorn	33-P9-4-5
Field corn	192-DAS2C595-95	Popcorn	36-P6-1
Field corn	193-DAS2C599-93	Popcorn	3-P9-4-6
Field corn	199-PRO23-245-1	Popcorn	40-P8-1-5-9
Field corn	206-CHZM13134-66-2	Popcorn	41-P1-19
Field corn	25-30F33-69H26.1	Popcorn	42-P18
Field corn	30-DKB747-43H17.4	Popcorn	46-P4-4
Field corn	31-CD303-89H4.2	Popcorn	47-P8-2-2-2
Field corn	38-AVANT-10H5.1	Popcorn	51-P8-2-MULT
Field corn	39-DKB747-41H17.3	Popcorn	54-GP10
Field corn	43-DKB350-76H30.1	Popcorn	58-P9-5-1
Field corn	49-AVANT-12H5.3	Popcorn	62-P8-2-2-5
Field corn	4-AG8080-7H3.1	Popcorn	64-P8-1-5-10
Field corn	56-DKB747-36H17.2	Popcorn	65-P9-1-2
Field corn	59-POP202-177.1	Popcorn	72-P7-4-5
Field corn	5-T5-AVANT-14H5.5	Popcorn	74-P9-12-1
Field corn	60-FLASH-20H11.1	Popcorn	81-T3-P9-3-2
Field corn	61-DAS422-80H31.2	Popcorn	8-P7-L7-1
Field corn	66-30-23	Popcorn	96-P19
Field corn	67-POP201-198.4		
Field corn	68-A2560-66H23.4		
Field corn	69-TORK-54H20.3		
Field corn	6-POP103-88.1		
Field corn	71-31-88		
Field corn	7-30F33-71H26.2		

Field corn	76-POP102-90.1
Field corn	77-29-14
Field corn	78-31-97
Field corn	79-POP202-88.2
Field corn	80-30-15
Field corn	82-POP201-192.1
Field corn	83-29-92
Field corn	84-POP203-51.2
Field corn	86-CML13
Field corn	88-POP103-80.5
Field corn	89-30-29
Field corn	90-POP103-81.4
Field corn	92-POP202-76.1
Field corn	97-DKB350-19H9.1
Field corn	98-AVANT-13H5.4
Field corn	99-W57

Supplementary Table 2. Characterization of the sites used in the evaluation of the 132 tropical maize lines in this study.

Traits[1]	Season 2018–2019		Season 2019	
	Low P	Normal P	Low P	Normal P
Sand (%)	79.68	78.01	79.11	79.01
Silt (%)	10.70	10.22	10.54	10.08
Clay (%)	9.62	11.77	10.35	10.91
pH (H_20)	4.7	5.60	4.80	5.80
K (cmolc dm^3)	17.79	24.33	17.99	25.63
Ca (cmolc dm^3)	3.86	5.18	3.97	5.66
Mg (cmolc dm^3)	2.01	2.12	1.97	2.33
Al (cmolc dm^3)	0.18	0.01	0.17	0.00
P (mg dm^3)	5.11	17.32	4.87	19.12
Organic matter (%)	3.53	2.97	3.51	2.87

[1] Chemical and physical analyses were performed using sample from the 0-20 cm soil.

Supplementary Table 3 QTNs associated with the 24 traits evaluated in 132 maize inbred lines under greenhouse and field conditions under low and normal phosphorus.

QTN[1]	Trait[2]	*Dataset*[3]	Chr	Position (bp)	QTN effect	LOD *score*	PVE (%)	MAF	Allele	Method[4]
Greenhouse										
S1_46130565	NR	LP_G	1	46,130,565	0,61 ~ 1,11	4,36 ~ 12,61	2,33 ~ 7,52	0,451	C	2, 4, 5
S1_274486912	NR	NP_G	1	274,486,912	0,85 ~ 3,34	6,06 ~ 19,28	0,87 ~ 10,81	0,288	A	3, 4, 5
S5_143821769*[#]	NR	NP_G	5	143,821,769	1,73 ~ 5,09	6,73 ~ 10,26	1,60 ~ 11,19	0,101	A	2, 3, 5
S6_38336881	NR	NP_G	6	38,336,881	-1,61 ~ -1,49	10,10 ~ 12,53	1,61 ~ 6,05	0,144	C	2, 4, 5
S7_4686573	NR	NP_G	7	4,686,573	-1,06 ~ -0,87	5,66 ~ 8,61	0,79 ~ 3,37	0,198	T	1, 4, 5
S7_100649505	NR	NP_G	7	100,649,505	-2,91 ~ -1,47	3,23 ~ 16,43	1,54 ~ 10,12	0,067	A	2, 4, 5
S8_146094515	PS	LP_G	8	146,094,515	-0,12 ~ -0,05	3,21 ~ 12,48	0,46 ~ 6,32	0,050	G	2, 4, 5
S1_1159069	PS	NP_G	1	1,159,069	-0,49 ~ -0,47	8,14 ~ 9,72	0,41 ~ 5,41	0,042	A	2, 4, 5
S2_95693385*	PS	NP_G	2	95,693,385	-0,33 ~ -0,12	3,14 ~ 7,75	0,12 ~ 11,14	0,258	C	1, 2, 3, 5
S3_156677296	PS	NP_G	3	156,677,296	0,27 ~ 0,40	3,99 ~ 7,54	0,33 ~ 6,25	0,076	C	2, 4, 5
S6_155005522*	PS	NP_G	6	155,005,522	-0,49 ~ -0,16	5,00 ~ 10,48	0,25 ~ 7,39	0,355	A	2, 3, 5
S7_25825683	PS	NP_G	7	25,825,683	0,08 ~ 0,24	3,05 ~ 5,29	0,04 ~ 4,86	0,181	G	1, 2, 5
S8_21392578	PS	NP_G	8	21,392,578	0,22 ~ 0,32	5,58 ~ 9,00	0,32 ~ 4,48	0,084	T	2, 4, 5
S1_194054469	PUE	LP_G	1	194,054,469	-229,87 ~ -77,60	6,76 ~ 8,70	1,45 ~ 9,47	0,285	G	1, 3, 5
S4_57399123	PUE	LP_G	4	57,399,123	58,22 ~ 203,45	4,62 ~ 6,77	0,95 ~ 8,99	0,489	G	1, 3, 5
S10_135071144	PUE	LP_G	10	135,071,144	-196,00 ~ -96,50	4,65 ~ 12,73	0,64 ~ 10,95	0,090	T	2, 4, 5
S4_1920423	PUE	NP_G	4	1,920,423	-8,03 ~ -7,08	3,77 ~ 6,68	2,00 ~ 2,57	0,211	C	2, 4, 5
S10_119011145	PUE	NP_G	10	119,011,145	-12,46 ~ -10,23	3,54 ~ 6,73	3,79 ~ 5,63	0,189	C	1, 2, 5
S6_140205291	PUpE	LP_G	6	140,205,291	-232,80 ~ -178,57	4,02 ~ 4,85	0,39 ~ 5,33	0,070	A	2, 4, 5
S9_62306047	PUpE	LP_G	9	62,306,047	-488,91 ~ -350,57	6,98 ~ 8,99	1,24 ~ 17,16	0,051	C	1, 2, 4, 5
S9_141732463	PUpE	LP_G	9	141,732,463	-255,34 ~ -208,14	4,67 ~ 5,97	0,62 ~ 7,25	0,080	C	2, 4, 5
S1_201662879	PUpE	NP_G	1	201,662,879	-46,10 ~ -41,22	4,60 ~ 5,62	2,46 ~ 4,27	0,211	G	2, 4, 5
S4_198262446	PUtE	LP_G	4	198,262,446	-0,102 ~ -0,04	6,86 ~ 28,04	0,93 ~ 5,93	0,070	G	2, 4, 5
S4_226520334	PUtE	LP_G	4	226,520,334	0,00 ~ 0,04	4,78 ~ 13,13	0,01 ~ 1,76	0,130	G	2, 4, 5
S1_1159069	PUtE	NP_G	1	1,159,069	-0,04 ~ -0,01	3,83 ~ 62,32	0,38 ~ 3,48	0,042	A	2, 4, 5
S3_48507935	PUtE	NP_G	3	48,507,935	-0,06 ~ -0,00	6,56 ~ 47,73	0,44 ~ 5,73	0,161	T	2, 3, 4
S3_128660576	PUtE	NP_G	3	128,660,576	-0,02 ~ -0,00	4,33 ~ 41,43	0,19 ~ 2,28	0,067	G	2, 4, 5
S5_171242155	PUtE	NP_G	5	171,242,155	-0,02 ~ -0,01	5,50 ~ 59,14	0,29 ~ 2,44	0,127	T	2, 4, 5

S6_78787352	PUtE	NP_G	6	78,787,352	0,00 ~ 0,02	6,16 ~ 34,46	0,21 ~ 2,96	0,093	T	2, 4, 5
S7_922076	PUtE	NP_G	7	922,076	-0,01 ~ -0,00	4,42 ~ 25,52	0,01 ~ 1,43	0,500	G	2, 4, 5
S8_118408026	PUtE	NP_G	8	118,408,026	-0,03 ~ -0,00	4,68 ~ 68,62	0,17 ~ 2,90	0,084	T	2, 4, 5

Supplementary Table 3 Continuation...

S1_46688892	RB	LP_G	1	46,688,892	-15,73 ~ -3,75	5,32 ~ 11,85	0,47 ~ 16,31	0,265	C	1, 2, 3, 4, 5
S2_10880952[#]	RB	LP_G	2	10,880,952	-7,56 ~ -4,74	3,27 ~ 12,74	0,73 ~ 4,33	0,081	G	1, 2, 4, 5
S3_7970405	RB	LP_G	3	7,970,405	-9,98 ~ -7,08	6,31 ~ 11,12	0,67 ~ 10,04	0,081	C	1, 2, 4, 5
S9_147539289	RB	LP_G	9	147,539,289	-12,74 ~ -2,95	3,02 ~ 3,27	1,35 ~ 6,29	0,122	G	1, 2, 3
S1_151079747	RB	NP_G	1	151,079,747	1,87 ~ 10,23	3,12 ~ 4,85	0,60 ~ 5,42	0,215	A	1, 3, 4, 5
S1_297994498	RB	NP_G	1	297,994,498	2,65 ~ 5,31	3,62 ~ 4,36	0,56 ~ 2,71	0,086	C	1, 2, 5
S3_153660781[#]	RB	NP_G	3	153,660,781	-3,98 ~ -2,97	6,06 ~ 8,03	1,95 ~ 3,63	0,241	G	1, 2, 5
S4_831081	RB	NP_G	4	831,081	4,77 ~ 5,52	7,01 ~ 14,29	5,69 ~ 7,52	0,279	G	2, 4, 5
S5_206261152[#]	RB	NP_G	5	206,261,152	-9,63 ~ -5,73	3,30 ~ 10,24	1,64 ~ 5,54	0,050	G	2, 4, 5
S5_212662145[#]	RB	NP_G	5	212,662,145	-5,37 ~ -3,64	3,50 ~ 11,80	2,22 ~ 4,58	0,163	T	1, 2, 4, 5
S5_2538913[#]	RB	NP_G	5	2,538,913	4,02 ~ 4,74	5,73 ~ 7,29	2,26 ~ 3,76	0,163	C	1, 2, 5
S5_4736803	RB	NP_G	5	4,736,803	-3,38 ~ -2,53	3,44 ~ 6,41	1,07 ~ 2,30	0,198	G	1, 2, 5
S10_119011145	RB	NP_G	10	119,011,145	-6,42 ~ -4,86	4,71 ~ 12,12	4,44 ~ 7,75	0,186	C	2, 4, 5
S1_298221121	RD	LP_G	1	298,221,121	-0,01 ~ -0,00	3,96 ~ 11,36	0,10 ~ 6,51	0,480	T	2, 4, 5
S5_202232006	RD	LP_G	5	202,232,006	0,00 ~ 0,01	3,85 ~ 16,08	0,73 ~ 2,06	0,068	C	2, 4, 5
S6_166011830	RD	LP_G	6	166,011,830	-0,01 ~ -0,00	4,65 ~ 11,94	0,01 ~ 2,20	0,058	A	2, 4, 5
S1_273189469	RD	NP_G	1	273,189,469	-0,01 ~ -0,00	3,59 ~ 12,71	1,02 ~ 4,72	0,228	T	2, 4, 5
S4_231949382	RD	NP_G	4	231,949,382	-0,01 ~ -0,01	4,34 ~ 8,79	2,01 ~ 4,38	0,203	G	2, 4, 5
S5_76418848	RD	NP_G	5	76,418,848	-0,03 ~ -0,00	3,29 ~ 5,55	0,76 ~ 6,13	0,194	A	2, 3, 5
S1_46130565	RL	LP_G	1	46,130,565	20,63 ~ 34,97	4,51 ~ 10,95	2,54 ~ 4,15	0,450	C	1, 4, 5
S4_198262446	RL	LP_G	4	198,262,446	-98,96 ~ -48,96	5,20 ~ 16,43	3,79 ~ 15,50	0,068	G	2, 4, 5
S5_97898187	RL	LP_G	5	97,898,187	20,35 ~ 21,37	4,19 ~ 4,77	0,77 ~ 2,03	0,264	A	2, 4, 5
S9_11783675	RL	LP_G	9	11,783,675	-51,19 ~ -38,40	4,31 ~ 6,88	0,79 ~ 4,14	0,068	C	2, 4, 5
S10_61903122	RL	LP_G	10	61,903,122	-39,68 ~ -26,65	3,92 ~ 5,33	0,81 ~ 3,75	0,107	T	2, 4, 5
S2_11554048[#]	RL	NP_G	2	11,554,048	-82,38 ~ -22,69	4,35 ~ 5,08	0,70 ~ 6,70	0,364	T	2, 3, 5
S1_46130565	RSA	LP_G	1	46,130,565	3,42 ~ 4,45	8,65 ~ 9,77	1,32 ~ 7,13	0,450	C	1, 2, 4, 5
S5_97898187	RSA	LP_G	5	97,898,187	2,51 ~ 3,65	4,84 ~ 7,24	0,84 ~ 3,76	0,264	A	2, 4, 5
S9_11783675	RSA	LP_G	9	11,783,675	-8,93 ~ -5,92	7,16 ~ 8,92	1,18 ~ 7,39	0,068	C	2, 4, 5

S1_40649707	RSA	NP_G	1	40,649,707	3,39 ~ 4,16	5,21 ~ 7,52	3,43 ~ 4,90	0,423	G	2, 4, 5
S3_225847941	RSA	NP_G	3	225,847,941	-6,70 ~ -4,63	4,88 ~ 9,39	2,73 ~ 5,73	0,118	G	2, 4, 5
S5_194306366	RSA	NP_G	5	194,306,366	-5,26 ~ -2,90	3,19 ~ 8,03	1,39 ~ 4,56	0,161	A	2, 4, 5
S5_212662145[#]	RSA	NP_G	5	212,662,145	-6,75 ~ -3,90	4,98 ~ 10,09	2,50 ~ 7,51	0,163	T	1, 2, 4, 5
S6_67420575	RSA	NP_G	6	67,420,575	-3,19 ~ -2,79	3,67 ~ 3,87	1,98 ~ 2,26	0,296	C	2, 4, 5
S2_45102754	RV	LP_G	2	45,102,754	0,00 ~ 0,12	4,34~ 10,55	0,01 ~ 12,00	0,117	C	2, 4, 5
**Supplementary Table 3** Continuation...										
S9_12276367	RV	LP_G	9	12,276,367	-0,08 ~ -0,04	4,12 ~ 6,91	0,24 ~ 3,93	0,078	A	2, 4, 5
S7_172249266	RV	NP_G	7	172,249,266	0,03 ~ 0,15	4,12 ~ 8,46	1,75 ~ 9,78	0,483	C	3, 4, 5
S1_278918194	SB	LP_G	1	278,918,194	8,62 ~ 10,14	11,00 ~ 12,41	6,32 ~ 8,44	0,450	A	2, 4, 5
S1_31254170	SB	LP_G	1	31,254,170	5,61 ~ 8,33	5,30 ~ 7,17	2,55 ~ 5,42	0,380	C	2, 4, 5
S1_82636210	SB	LP_G	1	82,636,210	6,66 ~ 9,69	3,99 ~ 7,59	2,25 ~ 4,59	0,180	C	2, 4, 5
S2_170934541	SB	LP_G	2	170,934,541	-13,24 ~ -10,73	7,15 ~ 9,96	4,17 ~ 6,35	0,122	G	1, 2, 4, 5
S10_119011145	SB	NP_G	10	119,011,145	-18,91 ~ -9,25	4,21 ~ 10,74	2,09 ~ 13,88	0,189	C	1, 2, 4, 5
S1_244847955[#]	TB	LP_G	1	244,847,955	-19,50 ~ -14,94	4,35 ~ 8,10	2,06 ~ 4,52	0,070	G	2, 4, 5
S2_170934541[#]	TB	LP_G	2	170,934,541	-14,81 ~ -13,91	6,27 ~ 7,32	1,88 ~ 4,51	0,120	G	2, 4, 5
S9_9307741	TB	LP_G	9	9,307,741	-29,51 ~ -8,9	3,70 ~ 7,28	0,88 ~ 5,69	0,160	C	2, 3, 5
S9_12017215	TB	LP_G	9	12,017,215	-28,20 ~ -10,27	3,36 ~ 9,23	1,30 ~ 6,74	0,173	G	1, 2, 3, 4, 5
S1_151079747	TB	NP_G	1	151,079,747	12,00 ~ 18,27	6,33 ~ 12,43	3,05 ~ 7,08	0,211	A	2, 4, 5
S3_225847941	TB	NP_G	3	225,847,941	-23,44 ~ -10,87	3,39 ~ 12,04	1,57 ~ 7,30	0,118	G	2, 4, 5
S10_119011145	TB	NP_G	10	119,011,145	-17,08 ~ -10,96	4,91 ~ 6,20	2,31 ~ 5,62	0,189	C	1, 2, 5
Field										
S1_295517245	ED	LP_18	1	295,517,245	-3,97 ~ -0,64	3,26 ~ 11,56	1,11 ~ 10,46	0,333	T	2, 3, 5
S1_268501337	ED	LP_19	1	268,501,337	0,61 ~ 0,87	3,16 ~ 5,72	1,04 ~ 2,08	0,182	G	1, 2, 4
S1_273365307	ED	LP_19	1	273,365,307	0,84 ~ 1,45	3,22 ~ 7,61	1,68 ~ 4,97	0,148	G	2, 4, 5
S1_290890953	ED	LP_19	1	290,890,953	0,69 ~ 2,55	3,77 ~ 6,90	2,08 ~ 6,98	0,349	C	1, 3, 5
S2_134524396	ED	LP_19	2	134,524,396	-3,32 ~ -1,25	6,45 ~ 10,99	3,70 ~ 7,91	0,150	T	1, 2, 3, 4, 5
S4_136990051	ED	LP_18	4	136,990,051	-1,75 ~ -0,53	3,66 ~ 7,96	0,77 ~ 2,07	0,341	T	2, 3, 5
S10_2994714	ED	LP_19	10	2,994,714	-0,89 ~ -0,76	5,05 ~ 5,39	2,37 ~ 3,24	0,320	T	2, 4, 5
S1_27897932	ED	NP_19	1	27,897,932	-1,99 ~ -1,26	3,77 ~ 7,09	1,51 ~ 3,76	0,071	C	2, 4, 5
S2_49789916	ED	NP_19	2	49,789,916	-3,66 ~ -1,13	6,36 ~ 13,35	3,69 ~ 9,57	0,277	T	2, 3, 4, 5
S3_185822669	ED	NP_18	3	185,822,669	-2,66 ~ -1,34	5,72 ~ 19,39	4,75 ~ 18,62	0,412	A	2, 4, 5

S4_170438381	ED	NP_19	4	170,438,381	-1,89 ~ -0,96	3,41 ~ 10,55	2,80 ~ 5,06	0,325	C	2, 3, 4, 5
S4_226997355	ED	NP_19	4	226,997,355	0,72 ~ 2,37	4,23 ~ 7,32	1,43 ~ 3,89	0,261	G	2, 3, 4
S7_113008994	ED	NP_19	7	113,008,994	0,99 ~ 2,95	4,00 ~ 11,45	2,11 ~ 5,70	0,182	T	2, 3, 4, 5
S7_7624808	ED	NP_C	7	7,624,808	0,88 ~ 1,15	7,19 ~ 8,53	2,50 ~ 4,28	0,157	G	1, 2, 5
S8_166171826	ED	NP_19	8	166,171,826	0,55 ~ 2,61	3,00 ~ 6,43	0,91 ~ 5,06	0,293	T	2, 3, 4
S10_129376513	ED	NP_19	10	129,376,513	1,01 ~ 2,79	4,84 ~ 9,87	3,05 ~ 5,76	0,298	T	1, 3, 4
S1_146214839	EH	LP_18	1	146,214,839	-4,52 ~ -1,93	3,66 ~ 6,06	1,93 ~ 2,64	0,315	A	1, 3, 4
S1_250251205	EH	LP_19	1	250,251,205	-2,46 ~ -2,02	4,15 ~ 7,70	1,15 ~ 4,77	0,409	C	1, 4, 5
S2_203741950	EH	LP_18	2	203,741,950	1,93 ~ 6,84	4,77 ~ 7,17	1,28 ~ 6,71	0,417	A	1, 3, 4, 5

Supplementary Table 3 Continuation...

S2_80076492*	EH	LP_19	2	80,076,492	-7,07 ~ -1,38	3,52 ~ 8,01	1,33 ~ 8,73	0,315	G	1, 2, 3, 4, 5
S4_17744548	EH	LP_C	4	17,744,548	1,94 ~ 2,89	5,01 ~ 14,10	2,20 ~ 4,87	0,110	T	1, 2, 4, 5
S5_173664450	EH	LP_19	5	173,664,450	-6,11 ~ -4,02	4,27 ~ 7,35	2,51 ~ 3,74	0,062	C	2, 4, 5
S5_178502126*	EH	LP_C	5	178,502,126	-5,28 ~ -1,13	3,96 ~ 12,87	1,70 ~ 9,27	0,378	C	1, 2, 3, 5
S9_155546945	EH	LP_C	9	155,546,945	1,39 ~ 2,48	3,09 ~ 4,82	1,65 ~ 2,21	0,255	G	2, 3, 5
S2_53578447	EH	NP_18	2	53,578,447	2,85 ~ 4,67	4,27 ~ 8,63	1,18 ~ 3,18	0,071	G	2, 4, 5
S2_175324915	EH	NP_C	2	175,324,915	1,13 ~ 4,57	3,48 ~ 7,48	1,70 ~ 6,90	0,433	A	1, 2, 3, 4, 5
S4_183789892	EH	NP_18	4	183,789,892	2,69 ~ 3,21	8,33 ~ 12,15	3,97 ~ 5,66	0,476	C	2, 4, 5
S5_181913284	EH	NP_18	5	181,913,284	-4,01 ~ -3,42	5,05 ~ 8,06	2,22 ~ 3,05	0,095	T	2, 4, 5
S5_22656703	EH	NP_18	5	22,656,703	-2,31 ~ -1,8	4,96 ~ 7,63	1,92 ~ 2,91	0,460	A	2, 4, 5
S5_93277248	EH	NP_18	5	93,277,248	-4,98 ~ -4,03	6,11 ~ 8,37	2,61 ~ 3,99	0,079	G	2, 4, 5
S6_68926335	EH	NP_19	6	68,926,335	-3,57 ~ -2,7	6,44 ~ 7,77	3,65 ~ 6,21	0,248	A	2, 4, 5
S7_8061099	EH	NP_18	7	8,061,099	-5,48 ~ -3,87	5,62 ~ 9,17	1,95 ~ 3,93	0,063	A	2, 4, 5
S9_127563945	EH	NP_19	9	127,563,945	2,21 ~ 3,65	3,33 ~ 5,60	1,74 ~ 4,74	0,162	G	2, 4, 5
S10_64644474	EH	NP_19	10	64,644,474	2,06 ~ 2,81	4,01 ~ 5,80	1,63 ~ 3,02	0,181	G	1, 2, 5
S10_81022340	EH	NP_C	10	81,022,340	1,52 ~ 2,72	5,11 ~ 9,17	2,09 ~ 6,22	0,196	G	1, 2, 4
S1_182142620	EL	LP_19	1	182,142,620	-0,84 ~ -0,48	3,15 ~ 9,75	3,90 ~ 5,86	0,187	G	2, 3, 5
S3_207156417	EL	LP_19	3	207,156,417	-0,52 ~ -0,46	4,95 ~ 6,50	3,37 ~ 4,25	0,125	T	2, 4, 5
S4_186450627	EL	LP_18	4	186,450,627	-0,83 ~ -0,25	3,38 ~ 5,43	0,90 ~ 4,23	0,302	A	2, 3, 4, 5
S4_104237007	EL	LP_C	4	104,237,007	-0,53 ~ -0,37	4,92 ~ 8,66	1,82 ~ 5,57	0,093	C	2, 4, 5
S4_237306135*	EL	LP_C	4	237,306,135	0,21 ~ 0,30	5,66 ~ 7,60	1,50 ~ 4,36	0,279	T	2, 4, 5
S5_23593212	EL	LP_C	5	23,593,212	0,26 ~ 0,34	3,07 ~ 8,38	1,79 ~ 3,10	0,124	T	2, 4, 5

S7_83084045	EL	LP_19	7	83,084,045	0,20 ~ 0,29	3,10 ~ 4,52	1,43 ~ 3,08	0,445	G	2, 4, 5
S9_70979188	EL	LP_C	9	70,979,188	0,41 ~ 0,52	7,31 ~ 9,71	2,40 ~ 5,76	0,100	G	2, 4, 5
S1_144086193	EL	NP_19	1	144,086,193	-1,84 ~ -0,46	3,95 ~ 4,48	2,33 ~ 9,23	0,087	C	2, 3, 5
S1_249766139	EL	NP_19	1	249,766,139	0,34 ~ 0,42	3,98 ~ 6,36	2,84 ~ 4,43	0,230	C	2, 4, 5
S1_26691938	EL	NP_C	1	26,691,938	-0,52 ~ -0,34	4,10 ~ 5,34	1,21 ~ 4,24	0,077	G	2, 4, 5
S4_186450627	EL	NP_18	4	186,450,627	-0,60 ~ -0,37	3,81 ~ 8,70	2,12 ~ 5,34	0,301	A	2, 3, 4, 5
S6_135401676	EL	NP_C	6	135,401,676	-0,31 ~ -0,20	3,60 ~ 6,16	0,94 ~ 2,08	0,116	T	2, 4, 5
S8_152253236	EL	NP_19	8	152,253,236	0,32 ~ 0,42	3,65 ~ 6,10	1,87 ~ 3,17	0,153	A	1, 4, 5
S2_26047828	HI	LP_18	2	26,047,828	3,88 ~ 7,36	5,15 ~ 10,43	2,01 ~ 7,24	0,156	C	2, 4, 5
S2_111173579*	HI	LP_19	2	111,173,579	-9,93 ~ -7,71	3,62 ~ 5,28	0,73 ~ 2,10	0,039	C	2, 4, 5
S2_46808444	HI	LP_C	2	46,808,444	-4,49 ~ -1,79	3,62 ~ 9,89	1,40 ~ 9,54	0,440	T	1, 2, 5
S3_203349832	HI	LP_18	3	203,349,832	-10,51 ~ -4,16	3,88 ~ 11,77	1,38 ~ 8,80	0,085	G	2, 4, 5

Supplementary Table 3 Continuation...

S3_165428591	HI	LP_C	3	165,428,591	1,92 ~ 3,94	3,73 ~ 7,67	1,21 ~ 5,06	0,220	A	1, 2, 4
S4_187030197	HI	LP_19	4	187,030,197	-15,55 ~ -3,4	5,98 ~ 8,39	0,98 ~ 9,79	0,290	C	1, 2, 3, 5
S8_148017105	HI	LP_19	8	148,017,105	3,03 ~ 10,42	3,52 ~ 6,12	0,97 ~ 5,29	0,484	C	2, 3, 5
S2_148344699	HI	NP_19	2	148,344,699	4,49 ~ 5,90	3,71 ~ 6,63	2,20 ~ 3,81	0,057	G	2, 4, 5
S2_211047798	HI	NP_19	2	211,047,798	3,35 ~ 4,30	5,45 ~ 9,30	3,11 ~ 5,13	0,163	T	2, 4, 5
S2_226288224	HI	NP_19	2	226,288,224	-3,60 ~ -2,3	3,57 ~ 6,94	2,17 ~ 5,17	0,270	T	2, 4, 5
S3_17190548	HI	NP_19	3	17,190,548	-6,88 ~ -2,0	3,84~ 6,81	1,88 ~ 5,19	0,319	C	2, 3, 5
S5_10594899	HI	NP_C	5	10,594,899	-6,50 ~ -6,06	6,38 ~ 9,59	4,88 ~ 5,62	0,077	C	2, 4, 5
S6_62712937	HI	NP_18	6	62,712,937	-5,98 ~ -4,62	4,95 ~ 6,38	2,82 ~ 4,69	0,238	G	2, 4, 5
S6_11431167	HI	NP_C	6	11,431,167	2,54 ~ 3,28	4,53 ~ 8,71	2,37 ~ 3,95	0,271	G	2, 4, 5
S7_165950985	HI	NP_19	7	165,950,985	1,80 ~ 4,89	3,29 ~ 4,34	1,61 ~ 2,97	0,434	A	2, 3, 5
S9_138041608	HI	NP_19	9	138,041,608	2,17 ~ 2,96	5,61 ~ 7,31	2,31 ~ 4,28	0,409	G	2, 4, 5
S9_147148478	HI	NP_19	9	147,148,478	-6,68 ~ -4,75	5,55 ~ 8,95	3,11 ~ 6,15	0,073	C	2, 4, 5
S10_127834511	HI	NP_C	10	127,834,511	5,18 ~ 6,38	8,69 ~ 8,95	5,12 ~ 7,77	0,116	C	2, 4, 5
S10_148468370*	HI	NP_C	10	148,468,370	-5,04 ~ -4,41	5,35 ~ 6,16	3,05 ~ 3,99	0,093	C	2, 4, 5
S1_278689620	PG	LP_18	1	278,689,620	-0,13 ~ -0,06	3,83 ~ 10,14	1,21 ~ 5,64	0,093	C	2, 4, 5
S1_173285277	PG	LP_19	1	173,285,277	-0,13 ~ -0,12	5,24 ~ 15,29	6,93 ~ 8,83	0,140	T	2, 4, 5
S3_44311414	PG	LP_18	3	44,311,414	-0,22 ~ -0,06	4,94 ~ 5,77	3,33 ~ 11,42	0,406	T	2, 3, 5
S3_205648132	PG	LP_C	3	205,648,132	-0,10 ~ -0,02	4,97 ~ 23,67	0,77 ~ 3,30	0,248	C	2, 3, 4

S6_163371503*	PG	LP_19	6	163,371,503	0,06 ~ 0,09	4,16 ~ 11,21	2,14 ~ 4,50	0,140	A	2, 4, 5
S8_15048496	PG	LP_18	8	15,048,496	-0,08 ~ -0,0	4,20 ~ 10,80	1,73 ~ 5,52	0,320	G	2, 4, 5
S8_146834197	PG	LP_19	8	146,834,197	0,10 ~ 0,16	5,04 ~ 8,73	2,69 ~ 5,96	0,062	A	2, 4, 5
S9_156510387	PG	LP_19	9	156,510,387	-0,08 ~ -0,08	3,69 ~ 8,52	2,85 ~ 2,93	0,125	G	2, 4, 5
S10_86299176	PG	LP_C	10	86,299,176	0,02 ~ 0,04	3,05 ~ 7,64	0,27 ~ 1,09	0,085	C	2, 4, 5
S1_259083391	PG	NP_18	1	259,083,391	-0,19 ~ -0,10	3,20 ~ 7,93	1,24 ~ 4,47	0,055	C	2, 4, 5
S1_165028962	PG	NP_C	1	165,028,962	-0,07 ~ -0,02	3,23 ~ 4,93	0,21 ~ 2,80	0,170	G	2, 4, 5
S2_37197043	PG	NP_18	2	37,197,043	-0,07 ~ -0,06	5,32 ~ 6,26	2,04 ~ 2,80	0,420	G	2, 4, 5
S3_105688175	PG	NP_18	3	105,688,175	0,08 ~ 0,26	4,61 ~ 6,55	2,38 ~ 6,48	0,198	A	2, 3, 5
S4_70379499	PG	NP_19	4	70,379,499	-0,13 ~ -0,10	3,24 ~ 5,37	0,64 ~ 1,92	0,082	C	2, 4, 5
S5_210099838	PG	NP_19	5	210,099,838	-0,11 ~ -0,08	3,36 ~ 5,34	0,52 ~ 1,86	0,090	G	2, 4, 5
S5_210099838	PG	NP_C	5	210,099,838	-0,08 ~ -0,00	3,41 ~ 7,73	0,01 ~ 2,39	0,085	G	2, 3, 4, 5
S7_71345290	PG	NP_19	7	71,345,290	-0,15 ~ -0,13	4,312 ~ 6,01	0,56 ~ 3,15	0,073	A	2, 4, 5
S7_157205983	PG	NP_C	7	157,205,983	-0,10 ~ -0,05	3,84 ~ 16,37	1,16 ~ 5,05	0,139	C	2, 4, 5
S1_217248337	PH	LP_18	1	217,248,337	-4,60 ~ -4,01	5,32 ~ 7,04	1,78 ~ 4,05	0,165	C	1, 2, 4, 5
**Supplementary Table 3** Continuation...										
S1_279964224	PH	LP_18	1	279,964,224	-9,16 ~ -2,69	3,93 ~ 9,56	2,37 ~ 6,87	0,410	C	2, 3, 4, 5
S1_33127662	PH	LP_19	1	33,127,662	-3,51 ~ -2,78	4,08 ~ 8,80	3,54 ~ 5,62	0,354	C	1, 2, 5
S1_153715305	PH	LP_19	1	153,715,305	0,00 ~ 0,02	4,44 ~ 31,80	0,59 ~ 9,31	0,328	C	2, 3, 4
S2_3581419	PH	LP_18	2	3,581,419	-6,55 ~ -5,88	7,24 ~ 12,53	2,67 ~ 5,22	0,118	C	1, 2, 5
S2_31500442	PH	LP_18	2	31,500,442	-7,32 ~ -2,92	3,85 ~ 12,77	1,75 ~ 10,99	0,181	A	1, 2, 4, 5
S2_128362461	PH	LP_18	2	128,362,461	3,05 ~ 4,16	4,69 ~ 11,21	1,80 ~ 4,37	0,244	C	1, 2, 4, 5
S2_89509296*	PH	LP_19	2	89,509,296	-5,02 ~ -3,30	4,10 ~ 6,33	1,95 ~ 4,50	0,100	C	2, 4, 5
S4_1253244	PH	LP_18	4	1,253,244	2,82 ~ 3,13	4,96 ~ 8,27	1,36 ~ 3,19	0,393	T	1, 2, 5
S1_259083391	PH	NP_18	1	259,083,391	3,41 ~ 5,39	3,82 ~ 5,12	1,34 ~ 3,35	0,131	C	2, 4, 5
S2_6154599	PH	NP_19	2	6,154,599	-8,09 ~ -2,51	3,83 ~ 6,41	1,72 ~ 4,43	0,248	T	2, 3, 5
S2_150779362	PH	NP_19	2	150,779,362	-12,91 ~ -3,78	5,38 ~ 7,10	2,50 ~ 7,27	0,139	C	2, 3, 4, 5
S5_210099838	PH	NP_C	5	210,099,838	3,06 ~ 3,91	4,29 ~ 12,27	5,08 ~ 8,28	0,325	G	2, 4, 5
S7_95115540	PH	NP_18	7	95,115,540	-6,14 ~ -1,93	3,04 ~ 13,47	0,94 ~ 9,51	0,480	G	2, 4, 5
S9_9646308	PH	NP_C	9	9,646,308	1,49 ~ 5,36	3,16 ~ 4,2	1,08 ~ 3,50	0,271	C	2, 3, 4
S10_139648418	PH	NP_19	10	139,648,418	-6,31 ~ -4,99	4,73 ~ 9,14	3,06 ~ 4,89	0,093	T	2, 4, 5
S10_147013208	PH	NP_19	10	147,013,208	3,27 ~ 6,86	3,04 ~ 7,11	3,13 ~ 4,59	0,279	C	2, 3, 4, 5

S10_147871005*	PH	NP_19	10	147,871,005	-3,55 ~ -2,43	4,01 ~ 5,54	2,15 ~ 4,57	0,480	T	1, 2, 5
S1_297589737	PHI	LP_C	1	297,589,737	0,00 ~ 0,00	4,12 ~ 10,49	0,16 ~ 0,68	0,062	T	2, 4, 5
S1_56967110	PHI	LP_C	1	56,967,110	-0,01 ~ -0,00	3,93 ~ 10,73	0,52 ~ 1,61	0,093	G	2, 4, 5
S2_8035127	PHI	LP_C	2	8,035,127	0,00 ~ 0,00	4,63 ~ 10,45	0,05 ~ 0,55	0,395	G	2, 4, 5
S5_195471833	PHI	LP_C	5	195,471,833	-0,01 ~ -0,00	7,69 ~ 10,61	1,06 ~ 2,62	0,193	A	2, 4, 5
S5_139942099*	PHI	LP_C	5	139,942,099	-0,01 ~ -0,00	9,97 ~ 11,11	1,01 ~ 3,25	0,186	G	2, 4, 5
S5_153897575	PHI	LP_C	5	153,897,575	0,00 ~ 0,01	8,65 ~ 11,77	1,12 ~ 3,56	0,263	A	2, 4, 5
S8_39678711	PHI	LP_C	8	39,678,711	-0,01 ~ -0,00	3,82 ~ 10,21	0,64 ~ 1,05	0,116	G	2, 4, 5
S9_113501158	PHI	LP_C	9	113,501,158	-0,01 ~ -0,00	4,41 ~ 10,49	0,00 ~ 2,32	0,201	G	2, 4, 5
S1_232380714	PHI	NP_C	1	232,380,714	0,00 ~ 0,00	6,54 ~ 99,01	0,01 ~ 1,15	0,426	G	2, 4, 5
S1_73129293	PHI	NP_C	1	73,129,293	-0,01 ~ -0,00	4,15 ~ 103,40	0,01 ~ 2,71	0,217	C	2, 4, 5
S1_41280339	PHI	NP_C	1	41,280,339	-0,01 ~ -0,01	8,96 ~ 97,68	0,65 ~ 3,02	0,077	G	2, 4, 5
S2_62413456	PHI	NP_19	2	62,413,456	0,00 ~ 0,01	3,99 ~ 34,14	0,00 ~ 2,75	0,090	C	2, 3, 5
S2_175324675	PHI	NP_C	2	175,324,675	-0,01 ~ -0,00	5,49 ~ 93,71	0,58 ~ 2,00	0,255	T	2, 4, 5
S4_176745674*	PHI	NP_19	4	176,745,674	0,00 ~ 0,01	3,15 ~ 15,72	0,10 ~ 0,29	0,073	G	2, 4, 5
S5_94820034	PHI	NP_18	5	94,820,034	-0,01 ~ -0,01	6,32 ~ 25,18	0,46 ~ 1,64	0,055	C	2, 4, 5
S5_3065314	PHI	NP_19	5	3,065,314	-0,02 ~ -0,01	3,33 ~ 28,64	0,27 ~ 2,49	0,057	A	2, 4, 5
S5_6813066	PHI	NP_19	5	6,813,066	0,01 ~ 0,01	8,05 ~ 30,81	0,91 ~ 1,38	0,123	A	2, 4, 5

Supplementary Table 3 Continuation...

S5_4222304	PHI	NP_C	5	4,222,304	0,00 ~ 0,01	5,47 ~ 90,30	0,83 ~ 1,84	0,116	G	2, 4, 5
S6_150946062*	PHI	NP_19	6	150,946,062	-0,01 ~ -0,00	9,01 ~ 12,26	0,67 ~ 1,09	0,204	A	2, 4, 5
S6_156249598*	PHI	NP_C	6	156,249,598	0,00~ 0,01	13,18 ~ 94,82	0,34 ~ 3,48	0,302	C	2, 4, 5
S7_6509723	PHI	NP_C	7	6,509,723	0,01 ~ 0,01	5,44 ~ 77,64	0,85 ~ 1,46	0,093	T	2, 4, 5
S9_140186211	PHI	NP_18	9	140,186,211	-0,01 ~ -0,00	7,44 ~ 20,31	0,28 ~ 2,37	0,214	A	2, 4, 5
S9_3310554	PHI	NP_19	9	3,310,554	-0,00 ~ -0,00	7,98 ~ 11,75	0,02 ~ 0,42	0,114	C	2, 4, 5
S9_13776063	PHI	NP_C	9	13,776,063	-0,01 ~ -0,01	11,02 ~ 93,33	1,14 ~ 2,54	0,085	G	2, 4, 5
S10_17562891	PHI	NP_19	10	17,562,891	-0,01 ~ -0,01	17,83 ~ 54,51	2,42 ~ 4,50	0,082	G	2, 4, 5
S1_51014576	PS	LP_19	1	51,014,576	0,04 ~ 0,05	4,11 ~ 14,19	1,16 ~ 4,67	0,279	G	2, 4, 5
S2_163217037	PS	LP_C	2	163,217,037	0,04 ~ 0,18	5,64 ~ 7,99	3,01 ~ 11,11	0,147	T	2, 3, 4, 5
S2_213193233	PS	LP_C	2	213,193,233	0,03 ~ 0,17	4,80 ~ 5,41	3,20 ~ 9,69	0,480	C	2, 3, 5
S3_191721493	PS	LP_18	3	191,721,493	0,07 ~ 0,08	3,81 ~ 5,10	1,79 ~ 2,45	0,108	G	2, 4, 5
S3_168278308	PS	LP_19	3	168,278,308	-0,16 ~ -0,07	3,29 ~ 19,61	4,22 ~ 11,78	0,356	C	2, 3, 4, 5

S4_197165902	PS	LP_C	4	197,165,902	0,01 ~ 0,08	3,29 ~ 8,54	0,20 ~ 3,92	0,062	G	2, 4, 5
S5_213813861*	PS	LP_18	5	213,813,861	0,04 ~ 0,11	3,16 ~ 8,04	1,75 ~ 3,07	0,356	A	2, 3, 5
S5_4039795	PS	LP_19	5	4,039,795	-0,08 ~ -0,04	3,26 ~ 7,86	1,52 ~ 3,44	0,100	C	2, 4, 5
S6_167023375	PS	LP_18	6	167,023,375	-0,17 ~ -0,05	3,35 ~ 6,16	2,20 ~ 4,79	0,209	G	2, 3, 5
S6_129844530	PS	LP_19	6	129,844,530	-0,11 ~ -0,08	7,14 ~ 13,86	3,50 ~ 4,80	0,100	C	2, 4, 5
S7_174193050	PS	LP_C	7	174,193,050	-0,05 ~ -0,04	3,52 ~ 7,45	2,11 ~ 3,44	0,116	A	2, 4, 5
S8_160939062	PS	LP_19	8	160,939,062	-0,11 ~ -0,08	6,73 ~ 11,50	2,53 ~ 3,28	0,062	C	2, 4, 5
S10_121486342	PS	LP_C	10	121,486,342	0,02 ~ 0,04	3,38 ~ 11,83	1,54 ~ 4,37	0,271	T	2, 4, 5
S2_47743618	PS	NP_18	2	47,743,618	0,09 ~ 0,15	4,10 ~ 8,00	2,30 ~ 5,69	0,095	T	2, 4, 5
S2_132827010	PS	NP_18	2	132,827,010	-0,27 ~ -0,01	3,10 ~ 5,74	0,08 ~ 5,21	0,111	A	2, 3, 4, 5
S2_12306582	PS	NP_19	2	12,306,582	-0,08 ~ -0,07	3,71 ~ 8,12	1,04 ~ 3,84	0,151	C	2, 4, 5
S3_168410267	PS	NP_18	3	168,410,267	0,07 ~ 0,20	5,17 ~ 6,72	2,99 ~ 5,96	0,269	G	2, 3, 4
S3_231459903	PS	NP_18	3	231,459,903	-0,13 ~ -0,07	3,59 ~ 10,76	1,86 ~ 6,25	0,158	C	2, 4, 5
S3_184664558	PS	NP_19	3	184,664,558	-0,07 ~ -0,05	3,38 ~ 7,05	0,55 ~ 4,73	0,218	T	2, 4, 5
S4_15064446	PS	NP_18	4	15,064,446	-0,26 ~ -0,05	3,02 ~ 7,63	1,50 ~ 8,17	0,214	A	2, 3, 4
S4_101953853	PS	NP_19	4	101,953,853	0,04 ~ 0,17	4,29 ~ 10,56	1,58 ~ 9,27	0,479	C	2, 3, 4, 5
S4_146635580	PS	NP_19	4	146,635,580	-0,33 ~ -0,10	7,18 ~ 14,37	1,12 ~ 10,63	0,084	T	2, 3, 4, 5
S5_19800950	PS	NP_C	5	19,800,950	-0,10 ~ -0,07	3,57 ~ 8,20	1,78 ~ 3,30	0,093	G	2, 4, 5
S10_2801162	PS	NP_19	10	2,801,162	-0,09 ~ -0,01	3,06 ~ 4,07	0,26 ~ 2,49	0,361	A	2, 3, 4
S1_246248875	PUE	LP_C	1	246,248,875	-5,47 ~ -1,49	3,81 ~ 7,01	1,52 ~ 5,08	0,139	C	3, 4, 5
S1_291977573	PUE	LP_C	1	291,977,573	1,81 ~ 3,65	3,50 ~ 12,36	4,64 ~ 8,06	0,480	G	2, 3, 4, 5

Supplementary Table 3 Continuation...

S2_32223271	PUE	LP_C	2	32,223,271	1,97 ~ 2,21	3,45 ~ 5,55	1,72 ~ 2,15	0,085	A	2, 4, 5
S3_190210784	PUE	LP_C	3	190,210,784	0,90 ~ 4,78	3,59 ~ 10,56	0,93 ~ 6,52	0,279	G	2, 3, 4, 5
S4_119430140	PUE	LP_19	4	119,430,140	-3,52 ~ -2,03	3,63 ~ 8,45	3,37 ~ 5,19	0,228	A	2, 3, 4
S6_119305424	PUE	LP_18	6	119,305,424	-10,29 ~ -6,68	9,40 ~ 16,4	1,97 ~ 12,41	0,070	A	2, 4, 5
S6_155082135*	PUE	LP_C	6	155,082,135	1,58 ~ 3,01	3,13 ~ 7,95	2,92 ~ 4,27	0,348	A	2, 3, 5
S7_111688769	PUE	LP_19	7	111,688,769	-5,76 ~ -1,51	3,52 ~ 7,37	0,58 ~ 6,23	0,144	G	1, 2, 3, 5
S8_148017105	PUE	LP_19	8	148,017,105	1,30 ~ 4,12	4,99 ~ 6,34	0,56 ~ 6,55	0,496	C	2, 3, 5
S5_10594899	PUE	NP_C	5	10,594,899	-0,97 ~ -0,70	6,64 ~ 7,00	2,46 ~ 4,81	0,077	C	2, 4, 5
S7_7624808	PUE	NP_C	7	7,624,808	0,50 ~ 0,91	4,44 ~ 10,44	2,29 ~ 7,83	0,155	G	2, 4, 5
S7_127897194	PUE	NP_C	7	127,897,194	0,33 ~ 0,66	3,58 ~ 4,33	1,21 ~ 4,91	0,196	A	1, 2, 5

S10_127834511	PUE	NP_18	10	127,834,511	0,88 ~ 1,03	4,57 ~ 6,44	1,00 ~ 2,52	0,111	C	2, 4, 5
S1_245295583	PUpE	LP_18	1	245,295,583	0,00 ~ 0,01	5,06 ~ 119,48	0,11 ~ 0,85	0,062	T	2, 4, 5
S1_44544531	PUpE	LP_18	1	44,544,531	0,00 ~ 0,01	9,19 ~ 110,44	0,25 ~ 2,56	0,328	T	2, 4, 5
S1_143952574	PUpE	LP_19	1	143,952,574	-0,00 ~ -0,00	9,72 ~ 113,29	0,04 ~ 0,41	0,062	C	2, 4, 5
S2_141296027	PUpE	LP_18	2	141,296,027	0,00 ~ 0,01	4,29 ~ 119,62	0,47 ~ 1,05	0,140	G	2, 4, 5
S3_31565213*	PUpE	LP_18	3	31,565,213	-0,012 ~ -0,00	7,88 ~ 111,68	0,04 ~ 1,55	0,070	C	2, 4, 5
S3_31565213*	PUpE	LP_C	3	31,565,213	-0,01 ~ -0,00	10,92 ~ 41,10	0,32 ~ 1,58	0,069	C	2, 4, 5
S3_190210784	PUpE	LP_C	3	190,210,784	0,00 ~ 0,00	3,13 ~ 61,52	0,56 ~ 0,80	0,279	G	2, 4, 5
S5_14070696	PUpE	LP_19	5	14,070,696	0,00 ~ 0,01	3,27 ~ 85,97	0,21 ~ 3,25	0,131	C	2, 3, 4, 5
S6_7139837	PUpE	LP_18	6	7,139,837	-0,01 ~ -0,01	25,80 ~ 98,64	1,16 ~ 3,89	0,492	G	2, 4, 5
S6_109559721	PUpE	LP_18	6	109,559,721	0,01 ~ 0,01	8,17 ~ 122,70	0,87 ~ 2,00	0,070	C	2, 4, 5
S6_7139837	PUpE	LP_C	6	7,139,837	-0,01 ~ -0,00	3,70 ~ 52,81	2,140 ~ 5,16	0,496	G	2, 3, 4, 5
S6_109559721	PUpE	LP_C	6	109,559,721	0,01 ~ 0,01	3,48 ~ 88,26	0,83 ~ 2,25	0,077	C	2, 3, 4, 5
S6_166375974	PUpE	LP_C	6	166,375,974	0,00 ~ 0,01	3,28 ~ 39,27	0,20 ~ 0,59	0,077	C	2, 4, 5
S8_171265799	PUpE	LP_18	8	171,265,799	-0,01 ~ -0,00	9,33 ~ 116,79	0,11 ~ 1,47	0,234	T	2, 4, 5
S8_19860789	PUpE	LP_19	8	19,860,789	-0,00 ~ -0,00	9,36 ~ 104,83	0,02 ~ 0,33	0,077	A	2, 4, 5
S9_131633080	PUpE	LP_C	9	131,633,080	0,00 ~ 0,00	4,45 ~ 14,35	0,03 ~ 0,31	0,085	C	2, 4, 5
S10_112468471	PUpE	LP_18	10	112,468,471	-0,01 ~ -0,01	6,61 ~ 119,67	0,75 ~ 1,11	0,109	T	2, 4, 5
S10_21019306	PUpE	LP_19	10	21,019,306	0,00 ~ 0,00	5,91 ~ 118,10	0,07 ~ 1,12	0,131	A	2, 3, 4, 5
S1_61943899	PUpE	NP_18	1	61,943,899	-0,00 ~ -0,00	16,74 ~ 20,51	0,03 ~ 3,49	0,388	G	2, 4, 5
S4_134429115	PUpE	NP_C	4	134,429,115	-0,00 ~ -0,00	7,54 ~ 94,16	1,53 ~ 1,92	0,100	G	2, 4, 5
S5_4020117	PUpE	NP_18	5	4,020,117	-0,00 ~ -0,00	14,73 ~ 64,29	1,71 ~ 5,13	0,452	G	2, 4, 5
S7_4256001	PUpE	NP_18	7	4,256,001	-0,00 ~ -0,00	13,71 ~ 82,83	1,39 ~ 3,31	0,127	C	2, 4, 5
S1_122231649	PUtE	LP_18	1	122,231,649	-42,70 ~ -25,72	3,89 ~ 6,79	1,76 ~ 3,59	0,062	G	2, 4, 5

Supplementary Table 3 Continuation...

S1_237149275	PUtE	LP_18	1	237,149,275	-34,75 ~ -28,39	3,15 ~ 4,89	1,64 ~ 2,83	0,054	G	2, 4, 5
S1_254528419	PUtE	LP_18	1	254,528,419	-20,87 ~ -18,01	4,65 ~ 7,21	2,45 ~ 4,19	0,304	G	2, 4, 5
S2_8919062	PUtE	LP_19	2	8,919,062	-35,95 ~ -25,27	4,72 ~ 6,81	1,16 ~ 3,03	0,156	G	2, 4, 5
S4_5071970	PUtE	LP_18	4	5,071,970	12,17 ~ 21,08	3,22 ~ 9,37	1,42 ~ 4,10	0,304	G	2, 4, 5
S4_85526697	PUtE	LP_18	4	85,526,697	-33,93 ~ -21,49	5,29 ~ 10,52	2,76 ~ 5,72	0,158	A	1, 2, 4 ,5
S4_167064633	PUtE	LP_19	4	167,064,633	-38,21 ~ -30,15	4,82 ~ 6,67	0,57 ~ 3,88	0,101	C	2, 4, 5
S4_77235640	PUtE	LP_C	4	77,235,640	-34,87 ~ -27,08	5,41 ~ 6,70	1,04 ~ 4,22	0,077	G	2, 4, 5

S5_36713069	PUtE	LP_18	5	36,713,069	-42,31 ~ -13,68	3,87 ~ 4,85	1,62 ~ 3,89	0,257	A	2, 3, 4
S7_134917410	PUtE	LP_18	7	134,917,410	-30,90 ~ -18,93	4,85 ~ 8,82	1,87 ~ 4,99	0,134	C	1, 2, 4
S8_62941084	PUtE	LP_C	8	62,941,084	-43,18 ~ -25,41	3,59 ~ 8,37	0,45 ~ 7,00	0,063	C	1, 4, 5
S10_137307447	PUtE	LP_19	10	137,307,447	-58,51 ~ -34,90	5,00 ~ 12,85	1,98 ~ 6,79	0,093	T	2, 4, 5
S1_92796935	PUtE	NP_18	1	92,796,935	-23,94 ~ -9,05	3,29 ~ 10,34	0,99 ~ 6,95	0,150	T	2, 4, 5
S1_154094903	PUtE	NP_19	1	154,094,903	15,81 ~ 23,58	5,06 ~ 11,91	2,75 ~ 6,11	0,427	A	2, 4, 5
S1_182384559	PUtE	NP_C	1	182,384,559	-33,64 ~ -13,22	3,24 ~ 11,13	2,32 ~ 5,27	0,133	G	1, 2, 3, 4, 5
S1_195303674	PUtE	NP_C	1	195,303,674	-37,58 ~ -8,77	3,34 ~ 14,44	1,26 ~ 9,94	0,173	A	1, 2, 3, 4, 5
S1_4262606	PUtE	NP_C	1	4,262,606	-28,39 ~ -22,04	3,58 ~ 9,28	2,89 ~ 4,10	0,054	T	2, 4, 5
S2_110719037*	PUtE	NP_C	2	110,719,037	-23,31 ~ -13,26	5,84 ~ 19,71	3,29 ~ 10,17	0,204	T	1, 2, 4
S7_141047027	PUtE	NP_18	7	141,047,027	-24,98 ~ -11,31	4,37 ~ 12,44	1,98 ~ 9,69	0,206	G	2, 4, 5
S7_6747669	PUtE	NP_19	7	6,747,669	-37,12 ~ -18,21	3,22 ~ 12,48	1,39 ~ 5,81	0,104	T	2, 4, 5
S3_67702517	SB	LP_18	3	67,702,517	-21,53 ~ -6,05	3,28 ~ 10,73	1,40 ~ 4,44	0,328	G	2, 3, 5
S3_67702517	SB	LP_19	3	67,702,517	-12,46 ~ -4,11	5,40 ~ 11,30	1,89 ~ 4,35	0,328	G	2, 3, 5
S3_67702517	SB	LP_C	3	67,702,517	-17,04 ~ -4,90	3,62 ~ 7,38	1,47 ~ 4,45	0,328	G	2, 3, 4, 5
S6_93657412	SB	LP_18	6	93,657,412	-14,61 ~ -13,78	5,01 ~ 5,43	1,48 ~ 3,38	0,101	T	2, 4, 5
S6_153605434*	SB	LP_18	6	153,605,434	6,167 ~ 23,9	3,36 ~ 5,15	0,62 ~ 4,76	0,257	C	2, 3, 5
S6_153605434*	SB	LP_19	6	153,605,434	3,97 ~ 15,65	3,74 ~ 7,45	1,54 ~ 5,97	0,257	C	2, 3, 4
S6_153605434*	SB	LP_C	6	153,605,434	4,00 ~ 17,74	3,01 ~ 5,47	0,40 ~ 4,18	0,257	C	2, 3, 4, 5
S8_155387189	SB	LP_19	8	155,387,189	-4,765 ~ -3,17	3,21 ~ 4,89	0,98 ~ 1,40	0,257	T	2, 4, 5
S10_140167314	SB	LP_18	10	140,167,314	-36,00 ~ -8,07	3,26 ~ 10,04	2,81 ~ 14,00	0,460	G	2, 3, 5
S10_140167314	SB	LP_19	10	140,167,314	-19,67 ~ -6,40	8,70 ~ 9,21	4,02 ~ 12,24	0,460	G	2, 3, 5
S10_140167314	SB	LP_C	10	140,167,314	-19,52 ~ -9,71	3,30 ~ 10,83	3,11 ~ 6,75	0,460	G	2, 3, 5
S1_187152852	SB	NP_19	1	187,152,852	-17,85 ~ -7,94	3,12 ~ 20,77	0,77 ~ 8,85	0,114	G	1, 2, 4, 5
S2_187701262	SB	NP_18	2	187,701,262	11,01 ~ 22,34	3,01 ~ 7,17	2,98 ~ 4,04	0,193	A	1, 3, 5
S4_6553058	SB	NP_18	4	6,553,058	9,70 ~ 16,04	4,08 ~ 7,75	2,16 ~ 5,91	0,177	G	1, 2, 4
S4_146471388	SB	NP_18	4	146,471,388	-13,86 ~ -11,79	6,11 ~ 9,55	3,73 ~ 5,68	0,250	C	1, 2, 5
**Supplementary Table 3** Continuation...										
S6_9197399	SB	NP_19	6	9,197,399	-9,42 ~ -6,62	5,60 ~ 8,47	2,22 ~ 5,69	0,368	G	1, 2, 4, 5
S7_21341225	SB	NP_C	7	21,341,225	-20,23 ~ -5,64	5,50 ~ 6,60	2,02 ~ 6,52	0,228	C	1, 3, 4
S9_37413046	SB	NP_18	9	37,413,046	10,22 ~ 18,23	3,00 ~ 5,57	0,75 ~ 3,15	0,063	A	2, 4, 5
S10_50838974	SB	NP_C	10	50,838,974	-23,79 ~ -5,42	4,00 ~ 6,73	1,52 ~ 7,32	0,170	A	2, 3, 4, 5

[1] QTNs identified highlighted with * and/or # are within meta-QTL regions identified by Zhang et al. (2014) or Guo et al. (2018), respectively

[2] Details about the traits are presented in Table 1

[3] 2018 season at low P (LP_18); 2018 season in normal P (NP_18); 2019 season at low P (LP_19); 2019 season in high P (NP_19); combination of 2018 and 2019 seasons in low P (LP_C); combination of 2018 and 2019 seasons in normal P (NP_C); greenhouse at low P (LP_G); and greenhouse at normal P (NP_G)

[4] 1 - mrMLM (Wang et al., 2016); 2 - FASTmrMLM (Tamba and Zhang, 2018); 3 - FASTmrEMMA (Wen et al., 2018); 4 - ISIS EM-BLASSO (Tamba et al., 2017); and 5 - pLARmEB (Zhang et al., 2017)

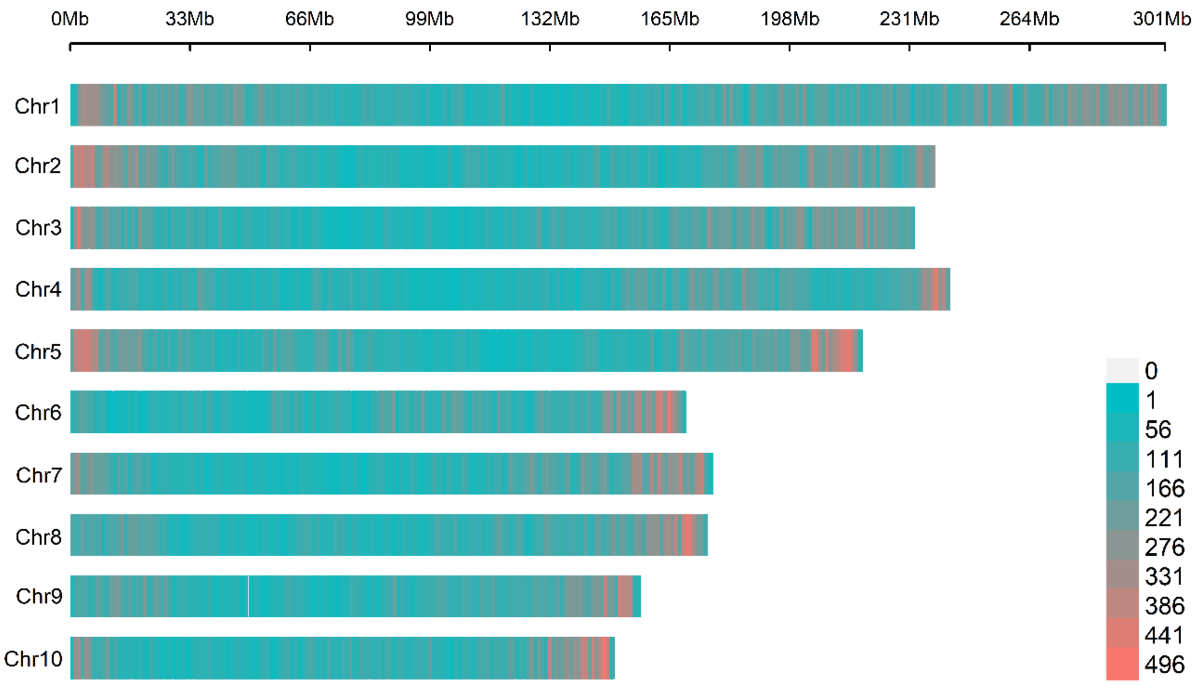

Supplementary Figure 1 Distribution of 273,775 SNP (Single Nucleotide Polymorphism) markers identified by genotyping-by-sequencing (GBS) along the ten chromosomes (Chr) of maize (*Zea mays* L.) in 1 Mb window size.

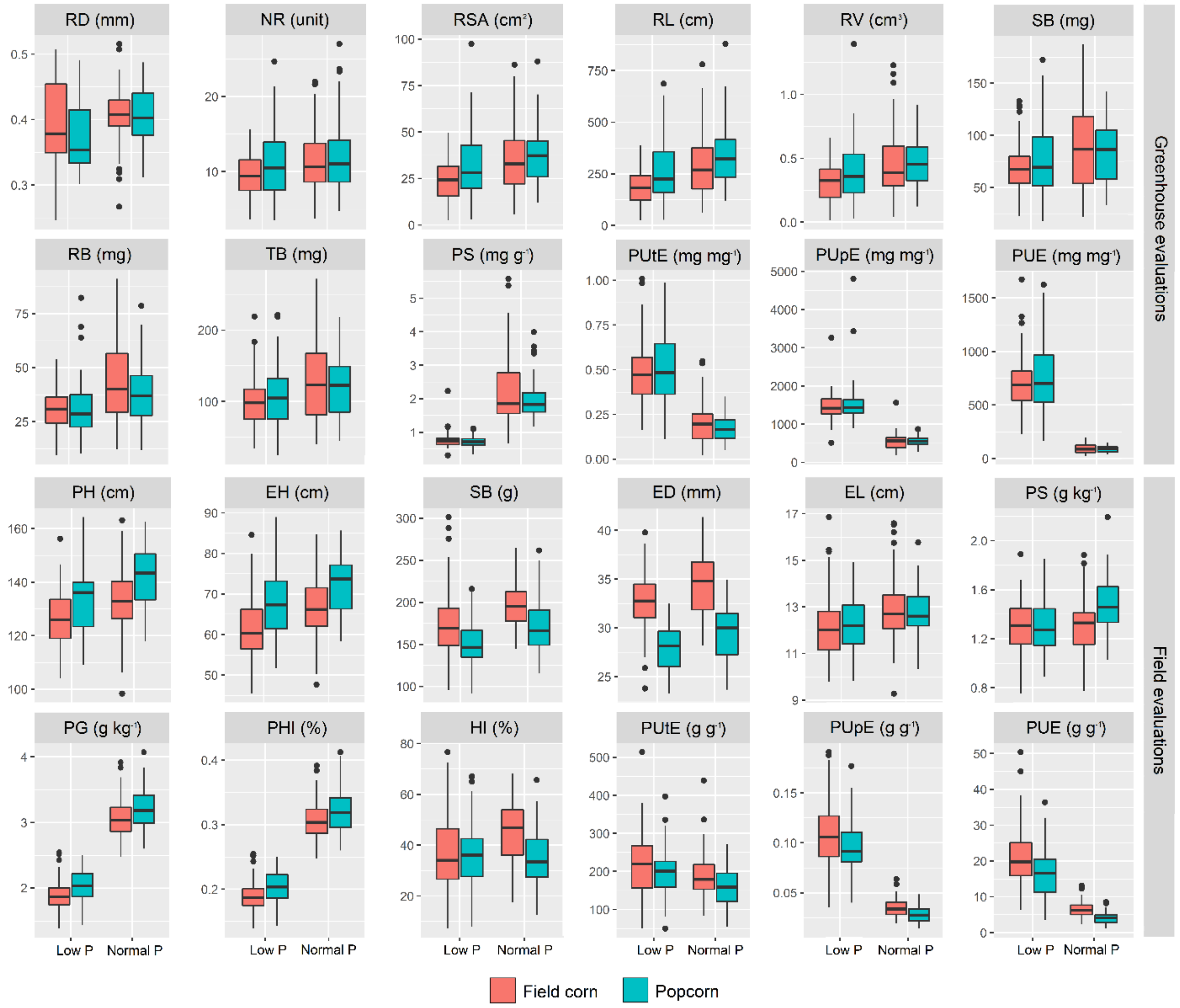

Supplementary Figure 2 Boxplots of the BLUP means of the 24 traits evaluated in 132 inbred maize lines evaluated under field and greenhouse conditions under low and normal P. The detailed list of traits is shown in Table 1.

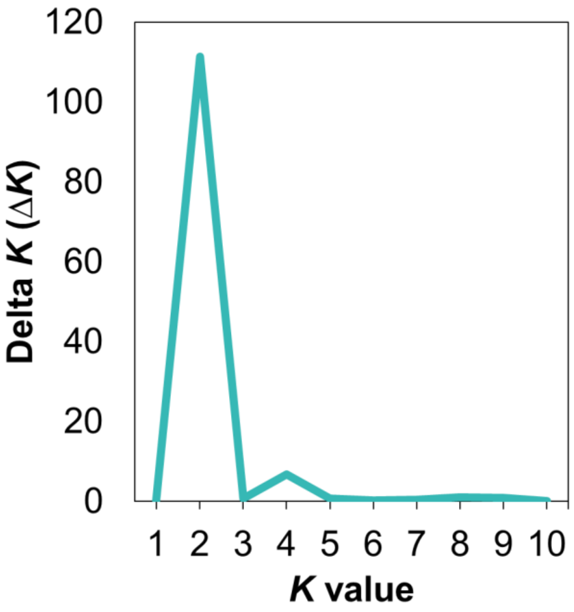

Supplementary Figure 3 Determination of genetically differentiated groups (K = 2) using the method described by Evanno et al. (2005). Bayesian cluster analysis was performed assuming mixed cluster (admixture) and correlated allelic frequencies.

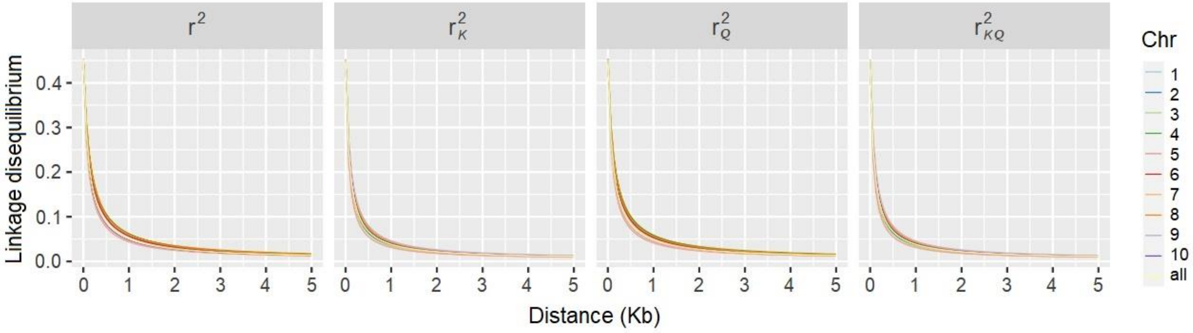

Supplementary Figure 4 Analysis of linkage disequilibrium decay (LD decay) according to physical distance using the conventional method (r^2), corrected for population structure (r^2K), kinship matrix (r^2Q), and population structure plus kinship matrix (r^2 KQ).

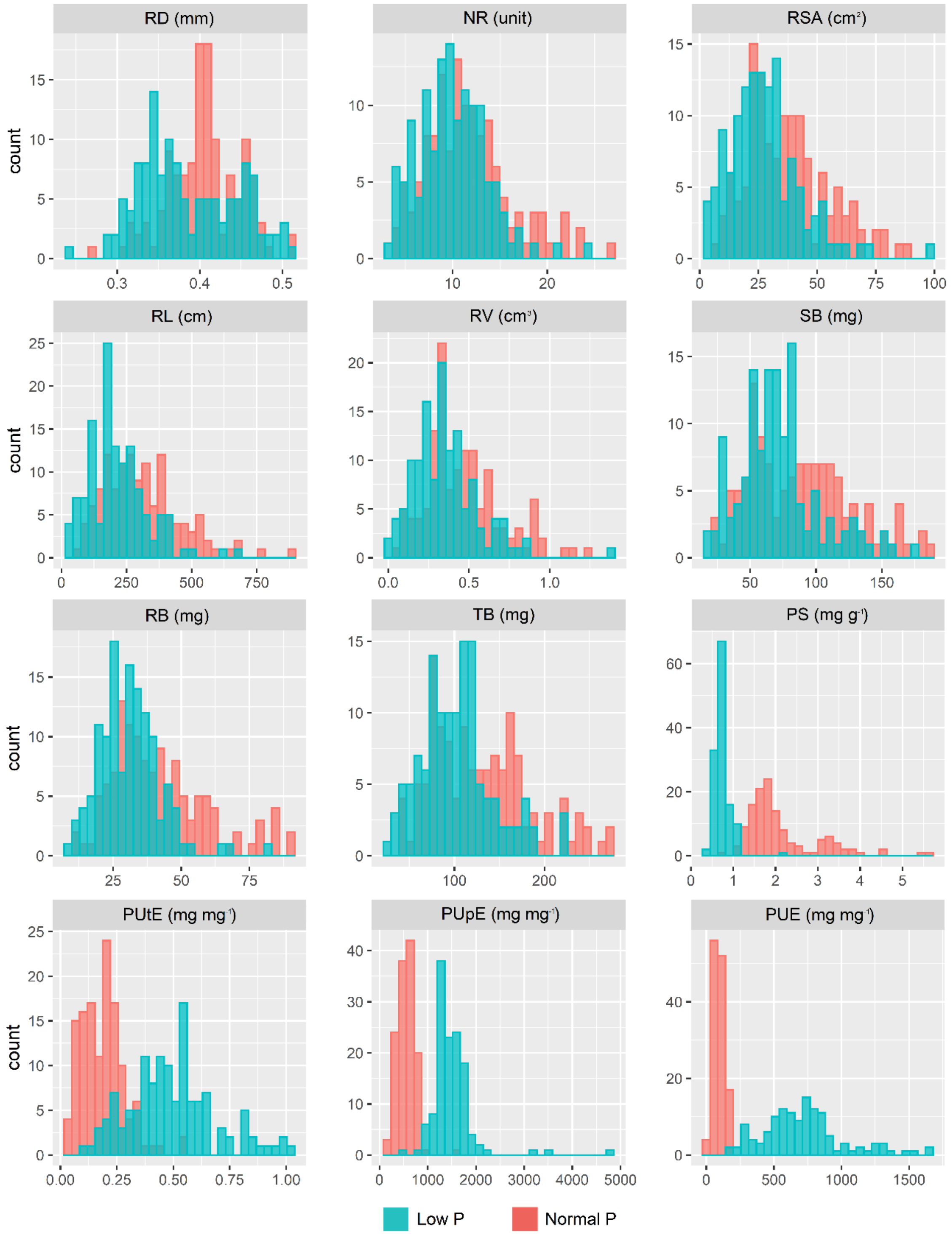

Supplementary Figure 5 Histograms of the least square means of 12 traits evaluated in 132 tropical maize lines under greenhouse conditions and hydroponic system. A detailed description of the evaluated traits is presented in Table 1

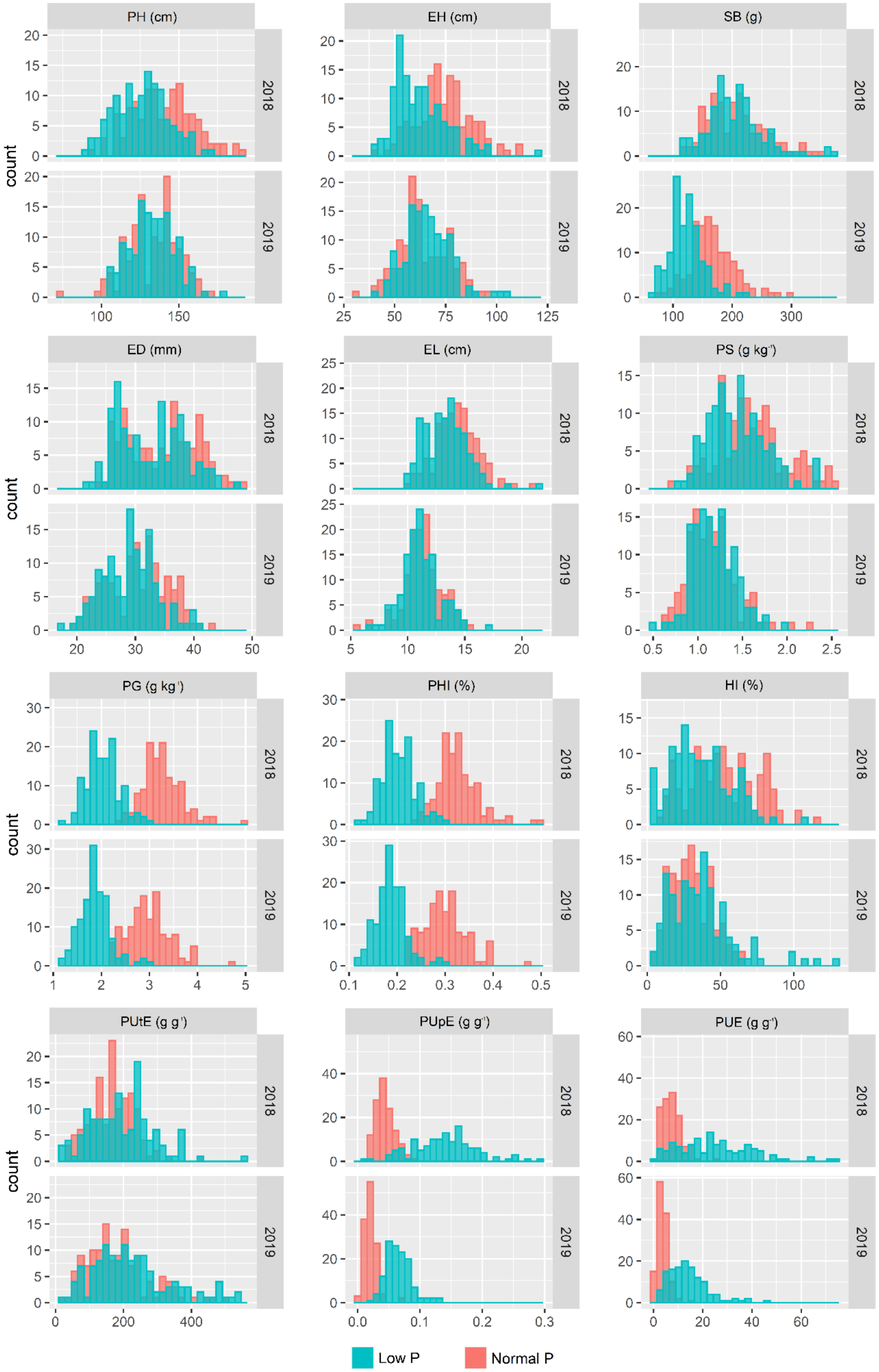

Supplementary Figure 6 Histograms of the least square means of 12 traits evaluated in 132 tropical maize lines in Londrina, Brazil, in the 2018 and 2019 seasons. A detailed description of the traits is presented in Table 1

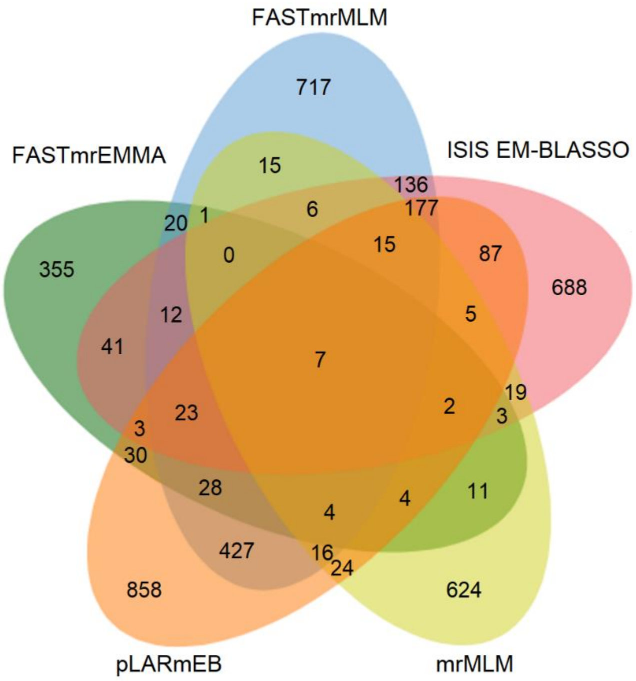

Supplementary Figure 7 Venn diagram of the distribution of the 5838 QTNs identified by the five different multi-locus GWAS methods evaluated.

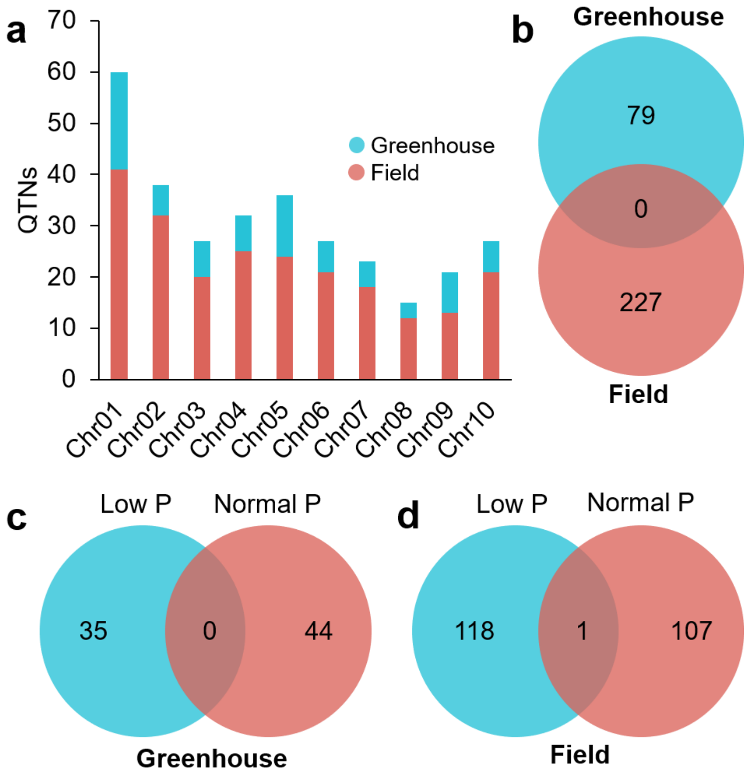

Supplementary Figure 8 Distribution of the 306 QTNs detected in the ten chromosomes (Chr) of maize (a), Venn diagrams of the number of QTNs identified between the greenhouse and field trials (b) and between low and normal P conditions in the greenhouse (c) and field (d).

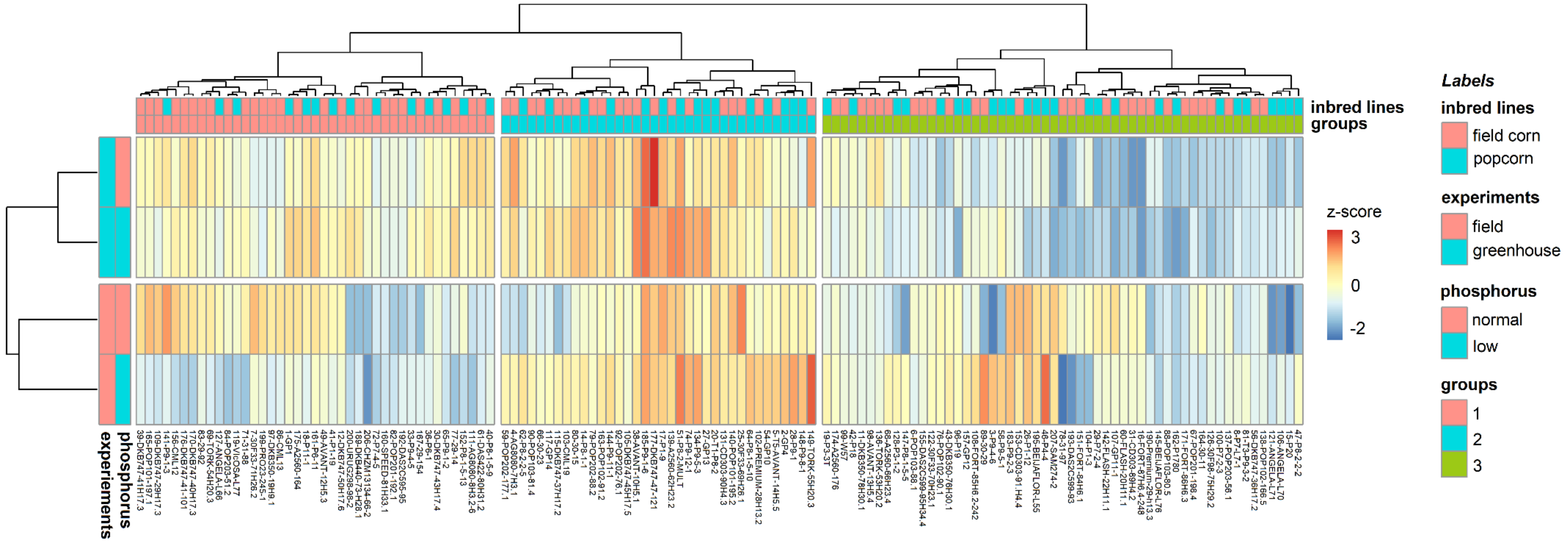

Supplementary Figure 9 Heatmap of the number of favorable alleles associated with traits related to phosphorus use efficiency in 132 inbred tropical maize lines evaluated in the greenhouse and field under low and normal P conditions.
